# In Vitro-Generated Hypertrophic-Like Adipocytes Displaying *PPARG* Isoforms Unbalance Recapitulate Adipocyte Dysfunctions In Vivo

**DOI:** 10.3390/cells9051284

**Published:** 2020-05-21

**Authors:** Marianna Aprile, Simona Cataldi, Caterina Perfetto, Maria Rosaria Ambrosio, Paola Italiani, Rosarita Tatè, Matthias Blüher, Alfredo Ciccodicola, Valerio Costa

**Affiliations:** 1 Institute of Genetics and Biophysics “Adriano Buzzati-Traverso,” CNR, Via P. Castellino 111, 80131 Naples, Italy; simona.cataldi@igb.cnr.it (S.C.); caterina.perfetto@igb.cnr.it (C.P.); rosarita.tate@igb.cnr.it (R.T.); alfredo.ciccodicola@igb.cnr.it (A.C.); 2 Department of Translational Medicine, University of Naples “Federico II” & URT “Genomic of Diabetes,” Institute of Experimental Endocrinology and Oncology “G. Salvatore,” CNR, Via Pansini 5, 80131 Naples, Italy; mariarosaria.ambrosio@unina.it; 3 Institute of Biochemistry and Cell Biology CNR, Via P. Castellino 111, 80131 Naples, Italy; p.italiani@ibp.cnr.it; 4 Department of Medicine, University of Leipzig, 4289 Leipzig, Germany; matthias.blueher@medizin.uni-leipzig.de; 5 Department of Science and Technology, University of Naples “Parthenope,” 80131 Naples, Italy

**Keywords:** hypertrophic adipocytes, *PPARG* isoforms, *PPARG* splicing, dominant-negative isoform, in vitro adipocytes, adipogenesis, hypertrophic obesity, insulin-resistance

## Abstract

Reduced neo-adipogenesis and dysfunctional lipid-overloaded adipocytes are hallmarks of hypertrophic obesity linked to insulin resistance. Identifying molecular features of hypertrophic adipocytes requires appropriate in vitro models. We describe the generation of a model of human hypertrophic-like adipocytes directly comparable to normal adipose cells and the pathologic evolution toward hypertrophic state. We generate in vitro hypertrophic cells from mature adipocytes, differentiated from human mesenchymal stem cells. Combining optical, confocal, and transmission electron microscopy with mRNA/protein quantification, we characterize this cellular model, confirming specific alterations also in subcutaneous adipose tissue. Specifically, we report the generation and morphological/molecular characterization of human normal and hypertrophic-like adipocytes. The latter displays altered morphology and unbalance between canonical and dominant negative (PPARGΔ5) transcripts of *PPARG*, paralleled by reduced expression of PPARγ targets, including *GLUT4*. Furthermore, the unbalance of PPARγ isoforms associates with *GLUT4* down-regulation in subcutaneous adipose tissue of individuals with overweight/obesity or impaired glucose tolerance/type 2 diabetes, but not with normal weight or glucose tolerance. In conclusion, the hypertrophic-like cells described herein are an innovative tool for studying molecular dysfunctions in hypertrophic obesity and the unbalance between PPARγ isoforms associates with down-regulation of *GLUT4* and other PPARγ targets, representing a new hallmark of hypertrophic adipocytes.

## 1. Introduction

The individual obesity-related risk for metabolic complications associates with storage capability of adipose tissue (AT). Energy buffering in the AT can occur either by tissue hyperplasia (i.e., de novo formation of new lipid-storing adipose cells) or hypertrophy of pre-existing adipocytes. According to the “overflow hypothesis”, exceeding the storage capability of adipose tissue leads to ectopic lipid accumulation, insulin resistance (IR), and type 2 diabetes (T2D) [1,2]. Consequently, similar metabolic consequences occur in conditions of deficiency and the excess of body fat, i.e., in lipodystrophies and obesity, respectively [3,4]. Particularly, hypertrophic obesity is associated with the reduced capacity to recruit and differentiate precursor cells into mature adipocytes [5,6,7,8]. Therefore, limited AT expandability, along with the balance between hyperplasia and hypertrophy, are key factors to clarify why not all obese individuals develop metabolic complications.

However, identifying the determinants accounting for the pathologic shift toward AT hypertrophy requires appropriate in vitro models able to recapitulate both the physiological processes governing adipocyte differentiation and the pathological causes of cells’ hypertrophy. In this regard, murine pre-adipocytes (i.e., 3T3-L1) have been widely used to study adipogenesis [9] as well as to generate hypertrophic cells in vitro [10]. Nevertheless, obvious differences between human and murine metabolism and physiology indicate the need to use more appropriate human models. Indeed, human primary pre-adipocytes [11,12,13] and adult mesenchymal stem cells—isolated from bone marrow, AT, umbilical cord and other tissues—represent the most reliable sources of cells able to differentiate toward the adipogenic lineage. The former cell type displays a proliferation/differentiation capacity that is strictly donor- and depot-related, showing unpredictable variability [11,14]. The latter displays low variability and high expansion/propagation capacity—especially for AT-derived cells—and are particularly useful for exploring early stages of differentiation, including the adipogenic commitment [15].

In this regard, we recently used a commercially available *hTERT*-immortalized cell line, i.e., AT-derived human mesenchymal stem cells (hMSCs), as model of human adipogenesis, in which we determined the negative impact of PPARγΔ5 isoform on PPARγ transcriptional activity and on adipocyte differentiation [16]. Together with the finding that PPARγΔ5 positively correlates with BMI and T2D [16], our results prompted us to evaluate whether the alteration of *PPARG* splicing is a feature of hypertrophic obesity.

Corroborating this hypothesis, our work reveals significant correlations between the expression of the different *PPARG* isoforms, subcutaneous adipocytes’ size and the inducible glucose transporter Glut4 (i.e., *SLC2A4* gene) in human subcutaneous adipose tissue (SAT). However, the intrinsic inter-individual variability and methodological issues related to adipocyte diameter calculation [17] represent sources of bias threatening the reliability and reproducibility of the results. Indeed, according to our previous study revealing highly variable PPARGΔ5 expression in human SAT, and considering the presence of complex feedback mechanisms regulating different *PPARG* isoforms [16,18,19], unpredictable genetic/environmental factors may affect *PPARG* expression and splicing in vivo. Therefore, it is glaring the need of a cellular model offering a direct comparison between normal and hypertrophic adipocytes and able to avoid—or at least reduce—any masking effect due to multiple unpredictable factors.

Thus, to recapitulate in vitro in a unique and highly reproducible model all the main molecular hallmarks of human hypertrophic AT, we setup a protocol for generating (for the first time, to the best of our knowledge) human hypertrophic-like adipocytes (HAs) that can be directly compared to mature cells (MAs) without confounding variables. Hence, in this work we report an accurate morphological, ultrastructural and transcriptional analysis of hMSCs differentiating into mature adipocytes, providing also evidence that the hypertrophic state associates with marked alterations in cell morphology, gene expression and *PPARG* splicing. This cellular model represents a versatile tool for studying structural remodeling and altered functionality of adipose cells during their pathologic evolution toward the hypertrophic state, as well as to test short- and long-term pharmacological treatments. Remarkably, analyzing this cellular model we confirmed that—similarly to large SAT adipocytes in vivo—hypertrophic-like cells display higher PPARGΔ5/cPPARG ratio and that such unbalance associates with marked deregulation in the network of *PPARG*-regulated genes, including those responsible of glucose transport and metabolism, insulin signaling and lipid droplet remodeling.

## 2. Materials and Methods

### 2.1. Human Samples

RNAs from subcutaneous adipose tissue biopsies were available in our laboratory from a previous study [16]. Samples were obtained from a clinically well-characterized German cohort of individuals (*n* = 94; mean age = 55.5 ± 16.5 y.o.; mean BMI = 35.4 ± 11.8) [20,21] undergoing bariatric surgery. The study was carried out in accordance with the Declaration of Helsinki, the Bioethics Convention (Oviedo), and EU Directive on Clinical Trials (Directive 2001/20/ EC) and approved by the University of Leipzig (approval numbers: 159-12-21052012 and 017-12-23012012). Random selection of samples, as well as exclusion criteria and classifications of individuals were applied as described in Aprile et al. (2018) [16]. Clinical and biochemical parameters were provided by Prof. Blüher’s unit, including visceral and subcutaneous mean and maximum diameter analyzed by Multisizer (Table 1).

### 2.2. Cell Lines and Cultures

*hTERT*-immortalized adipose derived mesenchymal stem cells (hMSCs) were purchased from American Type Culture Collection (ATCC SCRC-4000; Virginia, USA). Cells were cultured in DMEM-F12 (1:1) supplemented with 10% South American Fetal Bovine Serum (FBS), 2 mM glutamine, 30 units/mL penicillin, 30 mg/mL streptomycin, and maintained in humidified atmosphere of 5% CO_2_ at 37 °C. Media, sera, and antibiotics for cell culture were from Thermo Fisher Scientific (Waltham, MA, USA).

### 2.3. In Vitro Differentiation of Mature and Hypertrophic-Like Adipocytes

hMSCs were pulsed to differentiate in mature adipocytes as previously reported [16]. Briefly, cells between 5 and 12 passages have been plated at density of 3–4 × 10^3^/cm^2^ and induced toward adipocyte differentiation after reaching maximum confluence (48–72 h after plating). Cells at confluence were treated with Adipogenic Induction Mix (AIM; constituted by 850 nM insulin, 10 mM dexamethasone, 0.5 mM 3-isobutyl-1- methylXanthine, 33 mM biotin, 17 mM pantothenate, 1 mM rosiglitazone), and adipogenic maintaining mix (AMM; consisting of 850 nM insulin and 1 mM rosiglitazone). AIM and AMM were alternatively used for three days until 19–21 day, considered the terminal point of the process. Additionally, alternative differentiation protocols were tested adding the Bone Morphogenic Protein 4 (BMP4) bioactive protein (10, 20, and 50 ng/mL) to the AIM, or by using different FBS formulations (i.e., FBS qualified Australia and South American origin, Thermo Fisher Scientific, Waltham, MA, USA). Afterward, hypertrophic-like cells were obtained by treating mature adipocytes for 12 days with AMM mix supplemented with fatty acids i.e., palmitate, oleate, or both (350 μM). Such concentration reflects the pathological levels of fatty acids (200–375 μM) adipose cells of obese individuals are exposed to. Mixes were added to the cells every 3 days, and hypertrophic-like adipocytes were obtained within 32 days from adipogenesis induction.

### 2.4. Immunofluorescence Microscopy

For immunofluorescence analysis, hMSCs at different time points of adipocyte differentiation, grown on coverslips, were fixed with 4% formaldehyde for 15 min and washed in PBS. After washing, the cells were incubated with WGA 632/647 (red; 5 μg/mL) as membrane marker following manufacturer’s instructions. Afterward, cells were permeabilized with PBS/10% FBS/0.1% Triton X-100 for 5 min. Lipid droplets were marked with Bodipy 493/503 (green; 5 μL/mL) and cell nuclei were counterstained with DAPI (blue; 1 mg/mL). Reagents were purchased from Thermo Fisher Scientific (Waltham, MA, USA). Cells on coverslips were mounted with fluorescent mounting medium, and fluorescent labeling was examined using an A1 Resonance Plus confocal microscope (Nikon, Melville, NY, USA) and inverted (Leica DMI6000B) microscopy. Z-Stack imaging was performed by confocal microscopy for reconstruction of 3D images. Nikon Imaging Software (NIS) Elements Advanced Research software (version 4.50.00) was used for images acquisition/elaboration. All images were captured using a 20× Plan Apo lambda objective (1024 × 1024 pixels), numerical aperture 0.75, pinhole 1.2 AU, and exposure 6.2 s per pixel dwell. Detector sensitivity (gain) and laser power settings were kept the same for all collected images to allow for comparisons between images.

### 2.5. Cell Count and Oil Red O Staining for Quantifying Adipocyte Differentiation

Confocal microscopy images were processed by Image J [22] and analyzed for determining the percentage of differentiated cells. For each field, total cell number was determined by segmentation of nuclei stained with DAPI. Differentiated cells were identified and counted basing on the presence of lipid droplets stained with Bodipy 493/503. The percentage of cells that underwent adipogenic differentiation was calculated as cells positively stained with Bodipy ÷ the total number of cells (nuclei) × 100. Differentiation rate was calculated in five independent experiments and a total of ~6000 cells were analyzed. Additionally, adipocyte differentiation was estimated measuring lipid accumulation by Oil Red O staining [19,23]. Optical density determination at 510 nm was assessed by VICTOR Multilabel Plate Reader (Perkin Elmer, Massachusetts, USA), and the corrected subtracting background signal was determined by not specifically staining the undifferentiated cells.

### 2.6. Analysis of Adipocyte Size and Lipid Droplets

Cellular size and lipid droplet were analyzed in mature and hypertrophic-like adipocytes. Confocal Z-stack images were processed by open-source program Image J [22]. Adipocyte area was automatically measured after manual definition of cell perimeters. Lipid droplet number and size were analyzed by Image J macro “*MRI_Lipid_Droplets_Tool.ijm*,” which applies a Gaussian filter to the input images and an automatic threshold (percentile method) to remove artifacts from the mask of the droplets image, finally separating the touching droplets by a binary watershed transform. Therefore, starting from Z-stack images at focal plane with higher number of maximum diameters, lipid droplets marked with Bodipy were automatically selected and segmented by size threshold setting to 2 pixel/microns (expected size of smaller droplets). Multiple Z-stack images were used for reconstructing 3D projections, and for each cell/lipid droplet the focal plane with maximum size/diameter was considered. For lipid droplets analysis, individual parameters for accurate 3D surface selection were manually adjusted, increasing the accuracy of geometrical setting of touching droplets and background removal. Maximum diameter, related area and optical density were measured for each lipid droplet. Similarly, nuclei segmentation was applied for cell number counting, setting the size threshold to ≈50 pixel/microns. Such analysis was performed on both mature and hypertrophic-like adipocytes, calculating the average of total lipid area and the number of lipid droplets for cell and the mean area of a droplet.

### 2.7. Transmission Electron Microscopy

The hMSCs at different time points of adipocyte differentiation and upon the adipogenic induction were fixed in 2% glutaraldehyde, post-fixed in 1% osmium tetraoxide, dehydrated by being passed through a graded ethanol series, and embedded in Poly/Bed 812 resin (Polyscience, Warrington, PA, USA). The embedded samples were cut using a Leica ultracut UCT ultramicrotome (Leica Microsystems, Wetzlar, Germany) into ultrathin sections (50 nm thickness) and, to increase the contrast of the samples, an additional staining with uranyl acetate was performed. Finally, the samples attached to Formvar/carbon copper grids were observed under a model JEM-1011 (JEOL, Tokyo, Japan) transmission electron microscope using an accelerating voltage of 100 kV. Low- and high-magnification images were captured by iTEM software (Olympus Soft Imaging System, Münster, Germany). At least 10 different microscopic fields on multiple thin sections from different independent samples were observed and captured to obtain good confidence.

### 2.8. RNA Extraction, RT-PCR and qPCR

Total RNA was isolated using TRIzol Reagent (Life Technologies, Carlsband, CA, USA) according to manufacturer’s instructions. Quantification and purity of RNA was evaluated by NanoDrop spectrophotometer (Life Technologies, Carlsband, CA, USA). The synthesis of cDNA was performed with a High Capacity cDNA Reverse Transcription kit (Invitrogen, Carlsband, CA, USA) according to the manufacturer’s instructions. Expression analyses were performed by RT-PCR and qPCR techniques. Gene specific primers were designed using Oligo 4.0 program and listed in Table S1. RT-PCR products were amplified using MyTaq DNA Polymerase (Bioline, Memphis, Tennesse, USA) and analyzed by electrophoresis on agarose gel. PowerUp Sybr Green Master Mix (Thermo Fisher Scientific, Waltham, Massachusetts, USA) was used for qPCR expression analysis on CFX Connect Detection System (Bio-Rad, Hercules, CA, USA) according to manufacturer’s instructions. Relative mRNA expression was measured by 2^-ΔΔCt^ method. *PPIA* and *RPS23* were selected as housekeeping genes in hMSCs and SAT biopsies, respectively, evaluating the expression stability of at least three candidate housekeeping genes among *ACTB, HPRT, GAPDH, PPIA*, and *RPS23*. All reactions were performed in duplicates in at least three independent experiments.

### 2.9. Western Blot

Whole-cell lysates were obtained using RIPA lysis buffer supplemented with Halt Protease and Phosphatase Inhibitor Cocktail (Thermo Fisher Scientific, Waltham, Massachusetts, USA) and quantified by Bradford Assay Reagent (Bio-Rad, Hercules, CA, USA). For each sample, 40-60 mg of proteins were used for western blot analysis. According to manufacturer’s instructions, primary antibodies were used to different dilutions: anti-PPARγ (1:1000, Cell Signaling Technology, Danvers, Massachusetts, USA), anti-Glut4, anti-Adiponectin, anti-aP2, anti-Irs2 (1:500, Elabscience, Houston, Texas). Anti-Hsp90 (1:5000; Origene, Rockville, Maryland, USA) was used as a loading control antibody. Secondary anti-IgG (goat, mouse, and rabbit) antibodies were used at dilution 1:5000 (Bio-Rad, Hercules, CA, USA). Pierce ECL Western Blotting Substrate (Thermo Fisher Scientific, Waltham, Massachusetts, USA) was used for detection of immunoreactive bands. Quantification of protein levels (pixel density) was performed by *GelQuant.NET* software (www.biochemlabsolutions.com). Intensity values were normalized on Hsp90 expression and reference sample (i.e., the first time point having detectable levels).

### 2.10. Flow Cytometry Analysis

The expression of mesenchymal markers was analyzed by flow cytometry in hMSCs (T = 0 h), hMSCs-derived adipocytes (T = 20 day), and hypertrophic-like adipocytes (T = 32 day). The cells were incubated with PE-conjugated anti-CD73 antibody and FITC-conjugated anti-CD90 antibody, as well as with dye/isotype-matched antibodies (all from BD Biosciences, USA). The incubation was carried for 30 min at 4 °C in a dark environment. Afterward, unbound antibodies were washed out and the samples were processed by a BD FACS canto II (BD Biosciences, San Jose, CA) and analyzed using BD FACSDiva software. For each sample, 10^4^ events were acquired. Cells were counted and compared with the signals of the corresponding antibody isotype controls.

### 2.11. ELISA

Levels of the inflammatory cytokine IL-6 were determined by ELISA (R&D Systems, Minneapolis, USA) in cell-free supernatants according to the manufacturer’s instructions. Absorbance of assay wavelength was measured at 450 nm using a Cytation 3 imaging reader (BioTek, Winooski, VT, USA).

### 2.12. Quantification and Statistical Analysis

Student’s t test (one sample or two samples test; two tailed) was used for assessing statistical significance of differences in lipid accumulation (Oil Red O staining), cellular and lipid droplet area (3D analysis) between mature and hypertrophic-like adipocytes, as well as in gene expression assays (qPCR) for hMSCs at different stages of adipocyte differentiation. All assays were performed at least in triplicate. For each assay, the number of replicates, SD or SEM and statistical significance are reported in figure legends. The Kolmogorv–Smirnov test was used to analyze gene expression differences (qPCR) in SAT of patients. *p* value (*p*) ≤ 0.05 was considered significant. Statistical analysis of flow cytometry data was performed by BD FACSDiva software according to manufacturer’s instructions. Linear models were fit by *lm* function in R using the equation “result = lm(feature ~ cond, data, na.action = na.omit). For each specific analysis, *feature* was the response variable, *cond* was the regressor, and *data* was the dataframe containing expression data and clinical parameters. Missing fields (*na*) were omitted from regression. Residuals, Coefficients, Residual standard errors, Multiple R-squared, Adjusted R-squared, as well as F-statistic and *p*-values (ANOVA) were analyzed by the *summary* function. Pairwise correlations between couple of variables were carried out by the *cor* function in R language and Pearson’s (*r*) coefficient calculated as default parameter. Custom scripts in R language (using ggplot2) were used to generate the correlation, scatter, violin, and box plots.

Detailed information about all reagents and resources are provided in Table S2.

## 3. Results and Discussion

### 3.1. Unbalance of PPARG Isoforms in Patients with Hypertrophic Obesity

The subcutaneous adipose tissues of obese individuals and patients with T2D display reduced PPARγ activity [24] and increased relative amount of canonical and dominant negative transcripts (i.e., higher PPARGΔ5/cPPARG ratio) [16]. PPARGΔ5 mRNA levels positively correlate with BMI, and high levels of this dominant negative isoform reduce PPARγ transactivation ability and the adipogenic capacity of precursor cells [16]. Hence, considering that defective adipogenesis in adult AT and functional impairment of PPARγ are hallmarks of hypertrophic obesity, we decided to explore whether the pattern of *PPARG* splicing is modified in overweight and obese individuals, also considering the presence of related metabolic complications—i.e., the presence of impaired glucose tolerance (IGT) or T2D—and the adipocyte size.

**Figure 1:**
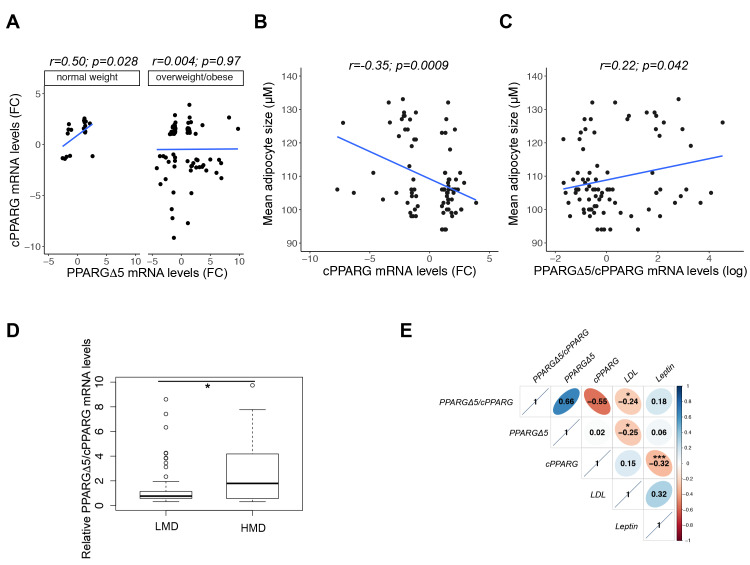
PPARGΔ5/cPPARG ratio correlates with adipocyte size, leptin and low density lipoprotein (LDL) cholesterol serum levels. Expression values of PPARGΔ5 and cPPARG have been previously measured in subcutaneous adipose tissue (SAT) of patients from a German cohort [16]. (**A**) Scatterplot reporting the correlation (linear regression analysis) between PPARGΔ5 and cPPARG expression levels in normal weight (*n* = 20) and overweight/obese individuals (*n* = 74). Pearson correlation coefficient (r) and *p* values (*p*) are shown. (**B**–**C**) Scatterplot resulting from regression analysis and indicating that cPPARG (B) and PPARGΔ5/cPPARG (C) levels oppositely correlate with mean diameter of subcutaneous adipocyte size (*n* = 86). Pearson correlation coefficient (r) and *p* values (*p*) are shown. (**D**) Boxplot showing PPARGΔ5/cPPARG levels in two subgroups, defined according to the mean diameter of subcutaneous adipocytes as “Low Mean Diameter” (LMD; mean diameter < 115μm, *n* = 63) and “High Mean Diameter” (HMD; mean diameter >115μm, *n* = 23) group. *p < 0.05. (**E**) Correlation plot indicating Pearson’s correlation coefficient and *p* value (* *p* < 0.05, *** *p* < 0.0001) among cPPARG, PPARGΔ5, PPARGΔ5/cPPARG, leptin (*n* = 61), and LDL serum levels (*n* = 59).

In this regard, we previously reported that PPARGΔ5 mRNA levels in the SAT are higher in overweight/obese than in individuals with normal weight [16]. Here, we observe that cPPARG and PPARGΔ5 mRNA levels are positively correlated between them only in the SAT of normal weight individuals (*n* = 20) and not in overweight/obese patients (*n* = 74, Figure 1A), suggesting that physiological balance between *PPARG* isoforms occurs exclusively in “healthy” AT. Indeed, our previous results indicated that *PPARG* splicing is modified in presence of metabolic disorders as obesity and T2D [16]. To address whether the unbalance between canonical and dominant negative *PPARG* isoforms associates with other relevant clinical parameters, we used PPARGΔ5 and cPPARG relative mRNA abundance from a previously analyzed German cohort [16] and compared their expression with respect to the size of adipocytes in the SAT (*n* = 86). Interestingly, cPPARG expression inversely correlates with the size of subcutaneous adipocytes (Figure 1B), whereas the ratio between the transcripts has a positive trend (Figure 1C, Figure S1A), compatible with the impaired metabolic profile of large adipocytes. Although it is not possible to define unhealthy hypertrophic adipose cells by means of their size, we used the mean diameter of subcutaneous adipocytes (115μm) to stratify patients in two groups—“Low Mean Diameter” (LMD; *n* = 63) and “High Mean Diameter” (HMD; *n* = 23)—in line with the work of Stenkula and Erlanson-Albertsson (2018) [17]. As shown in Figure 1D, PPARGΔ5/cPPARG ratio is higher in the SAT of HMD (vs. LMD), mostly because of a pronounced drop in cPPARG expression in this group (Figure S1B). These data provide evidence of *PPARG* isoforms unbalance in large adipocytes compared to small/medium-sized ones, suggesting a contribution of both *PPARG* expression and alternative splicing in compromising the metabolic homeostasis of these cells. Finally, extending the analysis to other clinical and biochemical parameters (Figure S1C), we disclosed that PPARGΔ5 negatively correlates with low-density lipoprotein (LDL) cholesterol levels, whereas cPPARG does not (Figure 1E). Conversely, only cPPARG negatively correlates with leptin serum levels (Figure 1E). Although these data support the unbalance of *PPARG* isoforms in the SAT of individuals with enlarged adipocytes, inter-individual variability, adipocyte heterogeneity in AT, or technical drawbacks (e.g., measurement of adipocyte size on histological specimen) could affect results’ consistency.

### 3.2. From Human Mesenchymal Stem Cells to Mature Adipocytes

**Figure 2:**
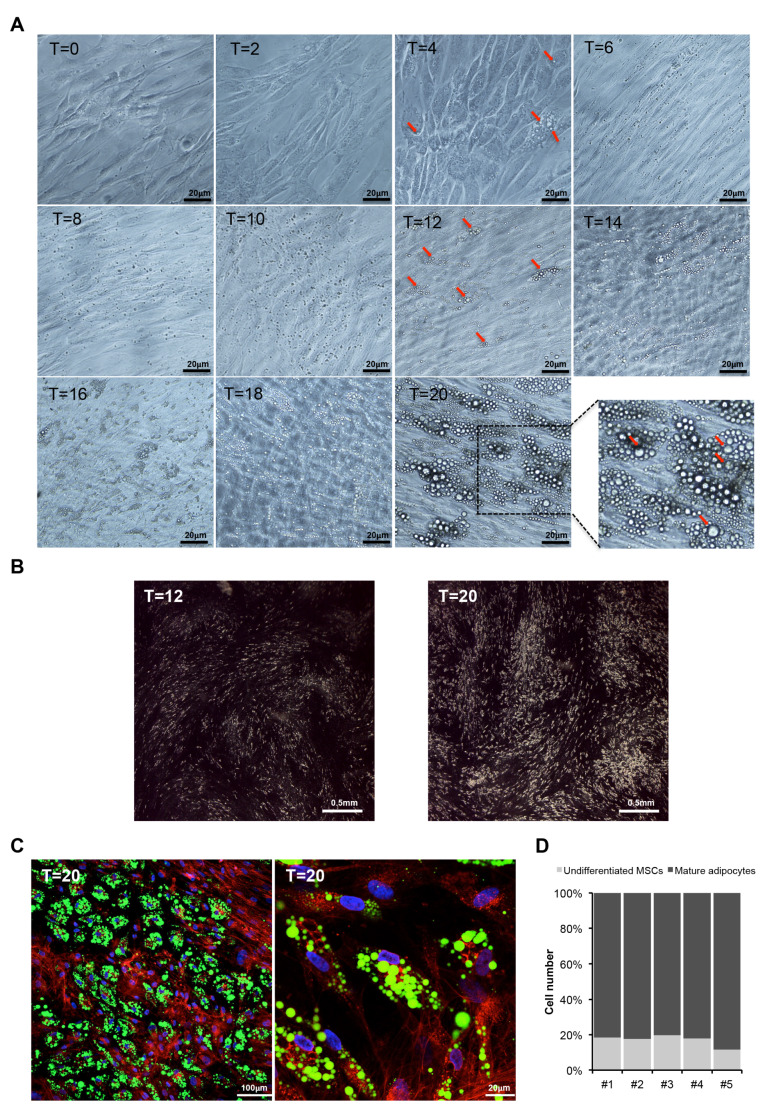
Human mesenchymal stem cells (hMSCs) are a reliable in vitro model of human adipocyte differentiation. (**A**) Representative phase-contrast images showing morphological changes of hMSCs along adipocyte differentiation, i.e., at starting point (T = 0 h), at different time points upon adipogenesis induction (T = 2, 4, 6, 8, 10, 12, 14, 16, 18 days) and at terminal differentiation (T = 20 days). Red arrows indicate some lipid droplets visible by optical microscopy during adipocyte differentiation (scale bar, 20 µm). (**B**) Representative images of hMSCs at 12 days and 20 days upon induction fixed with osmium tetraoxide and observed in dark field microscopy. The LDs are showed as white dots (scale bar, 1 mm). (**C**) Representative confocal microscopy images of hMSCs differentiated in mature adipocytes (T = 20 days): *nuclei* in blue (DAPI), lipid droplets in green (Bodipy 495/503) and cell membranes in red (WGA 632/647). Clusters of “bunch of grapes”–like droplets are evident (scale bar, 100 µm left panel; scale bar, 20 µm right panel). (**D**) Bar graph indicating the percentage of undifferentiated and differentiated cells in five independent experiments measured analyzing confocal images of hMSCs at terminal adipogenic differentiation (T = 20 days). Total number of cells was calculated counting nuclei stained with DAPI, differentiated cells were identified by positive staining of lipid droplets (Bodipy 495/503), and the number of undifferentiated hMSCs was calculated as the difference between total and differentiated cells. A total of ~6000 cells were analyzed.

To study *PPARG* splicing alteration in large dysfunctional adipose cells, in a more controlled and unbiased system, we set up a new model of human hypertrophic-like adipocytes, directly comparable to starting mature cells. Hence, we first assessed different experimental conditions for optimizing the differentiation protocols previously used [16,25]. In particular, after plating increased number of cells (Figure S2A) we confirmed that high densities favor adipocyte differentiation [26], whereas low cell densities, or high passage numbers, reduce the differentiation rate. As we previously described [16], using a modified version of the protocol reported by Janderová et al. (2003) [25], adipocyte differentiation of hMSCs is completely reached in 19–21 days, alternating two different mixes. Additionally, we did not measure significant increase in the differentiation rate neither supplementing cells with recombinant Bone Morphogenic Protein 4 (BMP4; Figure S2B) (capable of triggering commitment of MSCs into pre-adipocytes [27]) nor using different FBS formulations (data not shown). Up to day 2 after the adipogenic induction, hMSCs show the typical fibroblast-like shape (Figure 2A). Up to day 4 the cells maintain spindle-shape, even though cell circularity increases and immature droplets begin to be visible in the cytosol (Figure 2A). Therefore, according to the lipid droplets (LDs) formation model [28,29], this in vitro model requires about 4 days to complete triglyceride synthesis and to form oil lens within the endoplasmic reticulum and budding of lipid droplets into the cytosol. From day 4 to day 10, LDs number progressively increases and cells accentuate their characteristic whirlpool-like morphology. Afterward, both LDs number and volume markedly increase (Figure 2A,B). Particularly, on day 12, few clusters of “bunch of grapes”–like LDs became visible in a certain number of cells. Circularity is slightly emphasized, although cells maintain an elongated shape, similarly to mouse embryonic fibroblasts (MEFs) [30]. Around day 20, hMSCs appear evenly and appreciably differentiated into mature adipocytes, with an increased number of larger LDs and a marked whirling pattern with abundant clusters of “bunch of grapes”–like LDs (Figure 2A–C). In Figure 2B, hMSCs on day 12 and day 20—observed in dark field microscopy—show an increasing number of LDs and a typical swirled growth. Staining of neutral lipids combined to cell count by confocal microscopy (*n* > 6000) indicates that about 80% of hTERT-MSCs are terminally differentiated ~20 days upon induction (Figure 2D). Then, FACS analysis revealed a significant decrease of hMSCs surface markers CD73 and CD90 in mature adipose cells compared to undifferentiated precursors (Figure S3). These results, verified by several independent experiments, confirm the very low variability in the differentiation rate for this in vitro model.

### 3.3. From Mature to Hypertrophic Adipocytes

Adipocyte hypertrophy is a feature of dysfunctional AT and tightly associates with IR and T2D onset. Molecular mechanisms causing adipocytes’ dysfunctions in hypertrophic AT have not been completely clarified, and the cons and limitations of currently available in vitro models largely impede this task. Therefore, taking advantage of peculiar characteristics, such as the long propagation capacity, the good expansion and high population homogeneity of hMSCs, we set up a new protocol for generating in vitro human hypertrophic-like adipocytes—directly comparable to starting mature cells—that is useful to study the effects of adipocyte hypertrophy without confounding variables.

**Figure 3:**
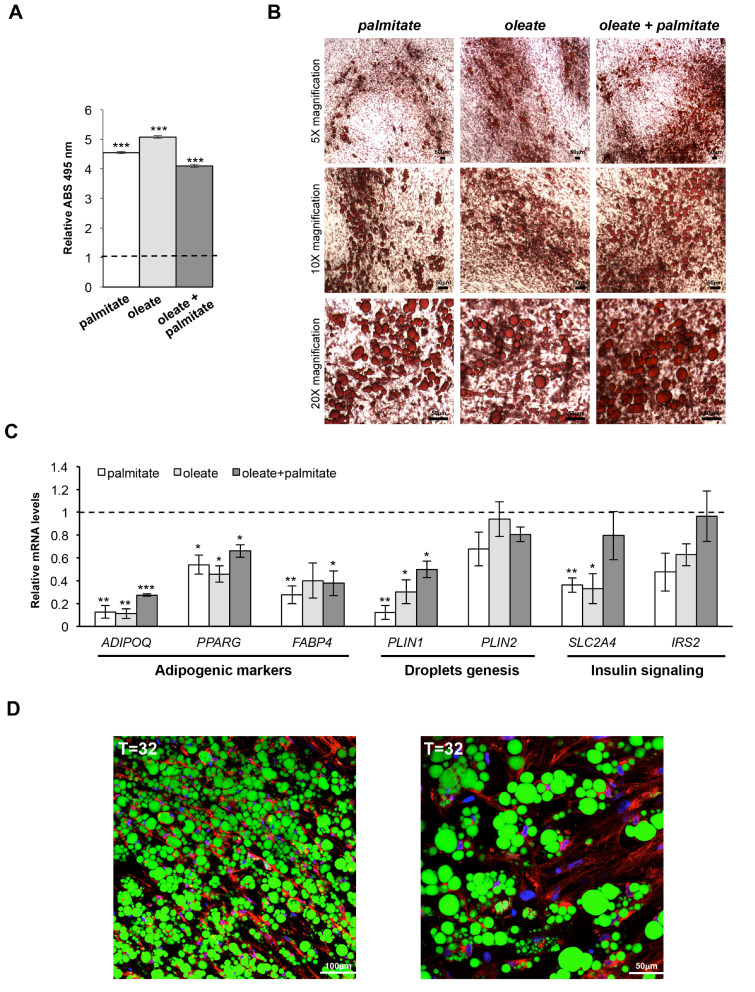
Hypertrophic-like cells generated from hMSCs-derived adipocytes. (**A**) Optical determination of lipid accumulation (Oil Red O staining) in hypertrophic-like adipose cells (HAs) generated from mature adipocytes (MAs)—differentiated in vitro by hMSCs—by supplementation of three different fatty acids mixes. Data are shown as mean ±SEM compared to mature adipocytes from three independent experiments. ***p val ≤ 0.001. (**B**) Representative bright-field images of HAs—generated by three different fatty acids mixes—after lipid droplets staining by Oil Red O (scale bar, 50 µm). (**C**) Relative mRNA quantification (qPCR) of *PPARG* and key target genes in HAs generated by three different treatments. *PPIA* was used as reference gene. Data are reported as mean ±SEM vs. mature adipocytes (dotted line) from three independent experiments. * *p* val ≤ 0.05, ** *p* val ≤ 0.01 and *** *p* val ≤ 0.001. (**D**) Representative confocal microscopy images of HAs (T = 32d) generated by palmitate-containing mix. Nuclei were stained by DAPI (blue), lipid droplets, and cell membranes by Bodipy 495/503 (green) and WGA 632/647 (red), respectively (scale bar, 100 µm left panel; scale bar, 50 µm right panel).

To this aim, hMSC-derived mature adipocytes were cultured with media containing saturated and/or monounsaturated fatty acids (MUFA) specifically selected for their high presence in human diet (i.e., palmitic and/or oleic acids), insulin and rosiglitazone for additional 12 days. Then, on day 32 (upon the adipogenic induction), cells supplemented with palmitate, oleate or their mixture were completely full of large LDs but no significant variation in lipid accumulation was measured among the different formulations of fatty acids (Figure 3A,B). Hence, to define which treatment induces the transcriptional alterations recapitulating at best the characteristics of hypertrophic adipocytes, we measured the expression of some selected genes. As shown in Figure 3C, HAs cultured in presence of palmitate (vs. fully differentiated MAs) revealed the most pronounced down-regulation of key adipogenic markers regulating AT homeostasis (*PPARG, ADIPOQ* and *FABP4)*, LDs biogenesis (*PLIN1* and *PLIN2*) and insulin signaling (*SLC2A4* and *IRS2*). Overall, these results indicate prolonged palmitate treatment as the most reliable way to induce hypertrophic-like features in MAs differentiated from hMSCs. Therefore, the above-mentioned protocol was chosen for in vitro generation of HAs. Additionally, FACS analysis revealed, as expected, a marked decrease of CD73 and CD90 in hypertrophic-like cells (Figure S3). As evident by confocal microscopy, treating hMSCs-derived MAs for additional 12 days (up to day 32) with insulin, PPARγ agonist (rosiglitazone) and palmitic acid induces visible LDs enlargement and the progressive formation of giant LDs (Figure 3D). Cell circularity substantially increases on day 32, and lipid-overburden leads to evident reduction of the cytoplasmic layer surrounding LDs with compressive effects on cell nuclei (Figure 3D, Figure S6A). Both hMSC-derived MAs and HAs can be further propagated and show a very weak susceptibility to dedifferentiate in vitro. Indeed, HAs (T = 32 day) cultured in standard growth medium show only a modest reduction of lipid content and mild morphological variations even when cultured for additional 30 days (i.e., 62 days after adipogenesis’ induction; Figure S4).

Therefore, the long propagation, culturing time, and durable cell attachment of MAs and HAs make hMSCs an advantageous human cellular model for studying in vitro physiologic adipogenesis—from very early to late differentiation stages—as well as pathologic conditions such as the hypertrophic state. Our results also indicate the possibility to expose cells to different stimuli and experimental conditions even for long time, without the risk of dedifferentiation and/or cell detachment. To the best of our knowledge, we describe the first in vitro-generated model of human hypertrophic-like adipocytes, directly comparable to control mature adipocytes. It is a suitable in vitro model for studying the molecular mechanisms causing the functional defects observed in the adipocytes of hypertrophic obese patients.

### 3.4. Stepwise Expression from hMSCs to Hypertrophic-Like Adipocytes

**Figure 4:**
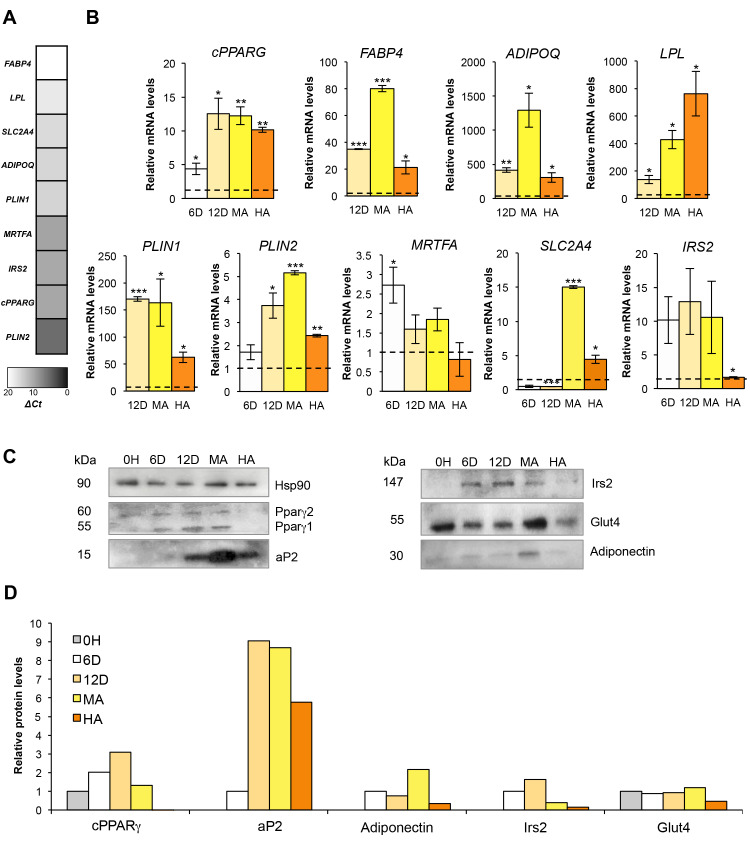
Expression analysis along differentiation of hMSCs in mature and hypertrophic-like adipocytes. (**A**) Gray-scale heatmap of normalized mRNA expression values (ΔCt = Ct gene – Ct *PPIA*) determined by qPCR in hMSCs (T = 0 h). *PPIA* was used as reference gene. (**B**) Relative mRNA expression analysis (qPCR) at different time points upon adipogenic induction (T = 0 h, T = 6 and T = 12 days) and in mature and hypertrophic-like adipocytes (MAs and HAs, respectively). For each gene, the first time point showing detectable levels was used as reference (dotted line; i.e., T = 0 h for *PPARG, PLIN2, MRTFA, SLC2A4, IRS2* genes; T = 6 days for *FABP4, ADIPOQ, LPL* and *PLIN1* genes). *PPIA* was used as reference gene in all assays. Data are reported as mean ±SEM from three independent experiments. *p val ≤ 0.05, **p val ≤ 0.01 and ***p val ≤ 0.001. (**C**) Western blots on lysates of hMSCs at different time points from adipogenic induction (T = 0 h, T = 6, and T = 12 days) and in MAs and HAs. Hsp90 was used as loading control. Representative autoradiographs are shown. (**D**) Bar graph reporting protein quantification (pixel density analysis of western blots). Values are normalized on Hsp90 (loading control) and—for each analyzed protein—the first time point having detectable levels (by the specific Ab) was used as reference (relative protein levels = 1).

The transcription factor PPARγ—master regulator of adipogenesis—belongs to the “second wave” of adipogenic factors, and therefore, it is expressed later than other factors in the adipogenic process [31]. Unexpectedly, undifferentiated cells show moderate basal expression of all *PPARG* transcripts, including the dominant negative PPARGΔ5 (Figure 4A, Figure S5A). Similar to our previous finding in human primary adipocyte precursor cells [19], also hMSCs display higher PPARG1 than PPARG2 mRNA basal levels (Figure S5A). However, *PPARG* expression progressively increases, reaching its highest levels 12 days after adipogenesis induction (Figure 4B), according to our previous analysis [16]. In line with PPARγ levels, its direct targets *FABP4*, *LPL*, and *ADIPOQ* [32,33] have almost undetectable levels in hMSCs (Figure 4A) and are markedly induced from day 6 up to terminal differentiation both at mRNA and protein level (Figure 4B–D).

Genes encoding perilipins—proteins involved in lipid storage and droplet biogenesis—show a peculiar expression pattern. As shown in Figure 4A and Figure S5B, *PLIN1* mRNA cannot be detected in undifferentiated hMSCs, whereas *PLIN2* gene (encoding the adipose differentiation-related protein, ADRP) is highly expressed, in line with the notion that ADRP locates on small LDs even in early differentiating cells and in 3T3-L1 murine pre-adipocytes [34,35]. Moreover, in line with previous analyses indicating that perilipin 1 is transcriptionally regulated by PPARγ and has AT-specific expression [34,35,36,37], *PLIN1* is strongly and rapidly induced upon stimulation with the adipogenic mix and shows the same trend of expression of other PPARγ target genes (Figure 4B, Figure S5B–D). *PLIN2* mRNA is modestly induced along hMSCs adipogenic differentiation and, starting from day 6, a progressive switch in the expression of perilipins occurs (Figure 4B, Figure S5B–D), as also reported for mouse embryonic fibroblasts (MEFs) and stromal vascular cells [38]. This finding suggests that perilipin 1 fully replaces ADRP on the surface of mature LDs, according to the hypothesized role of *PLIN1* in the formation of large LDs [39].

As previously described, adipocyte differentiation associates with profound changes of cell morphology [40]. In mouse pre-adipocytes, the interaction of monomeric G-actin with the transcriptional co-activator myocardin-related transcription factor A (MRTFA) prevents its nuclear translocation, inducing *Pparg* expression [41]. Interestingly, we observed an opposite trend of *MRTFA* gene expression compared to *PPARG* up to terminal differentiation (Figure S5E) suggesting that—also in the context of human neo-adipogenesis—MRTFA and PPARγ act in a mutually antagonistic manner.

Insulin resistance is hallmark of hypertrophic obesity [42,43,44], and PPARγ activation in adipose cells is sufficient to improve insulin sensitivity [45]. Among the genes involved in glucose uptake and insulin signaling, we measured *SLC2A4* (alias *GLUT4*) and *IRS2* expression since they are validated PPARγ targets [45,46]. As shown in Figure 4B, *SLC2A4* levels are higher in terminally differentiated adipocytes, whereas *IRS2* expression peaks on day 12, as also corroborated by protein quantification analysis performed along the entire adipogenic process (Figure 4C,D).

### 3.5. Hypertrophic-Like Adipocytes Display Morphological Features Resembling Adipose Tissue Hypertrophy

**Figure 5:**
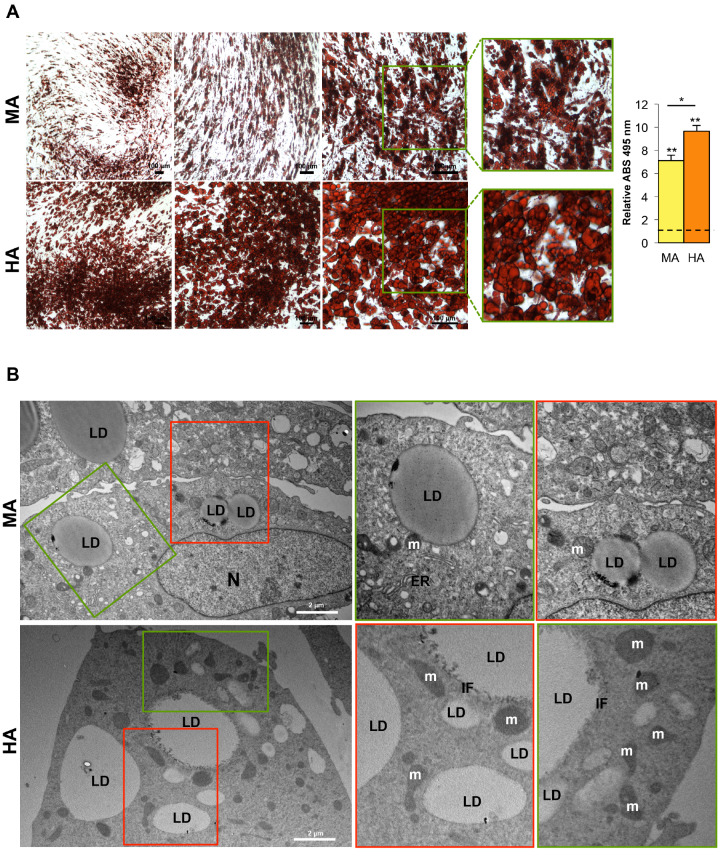
Mature and hypertrophic-like adipocytes display morphological differences. (**A**) Representative images (left panel) of mature and hypertrophic-like adipocytes (MAs and HAs, respectively) stained with Oil Red O (scale bars, 100 µm). Measurement of lipid accumulation (optical density) in hMSCs (T = 0 h used as control, CTR), in MAs and in HAs. Data are shown as mean ± SEM vs. CTR (dotted line). *p val ≤ 0.05 and **p val ≤ 0.01. (**B**) Representative micrographs of MAs and HAs by transmission electron microscopy (scale bars, 2 µm). Green and red squares in each left panel are observed at higher magnification in right panels. *n* = nucleus; m = mitochondria, LD = lipid droplets, ER = endoplasmic reticulum, IF = intermediate filaments.

**Figure 6:**
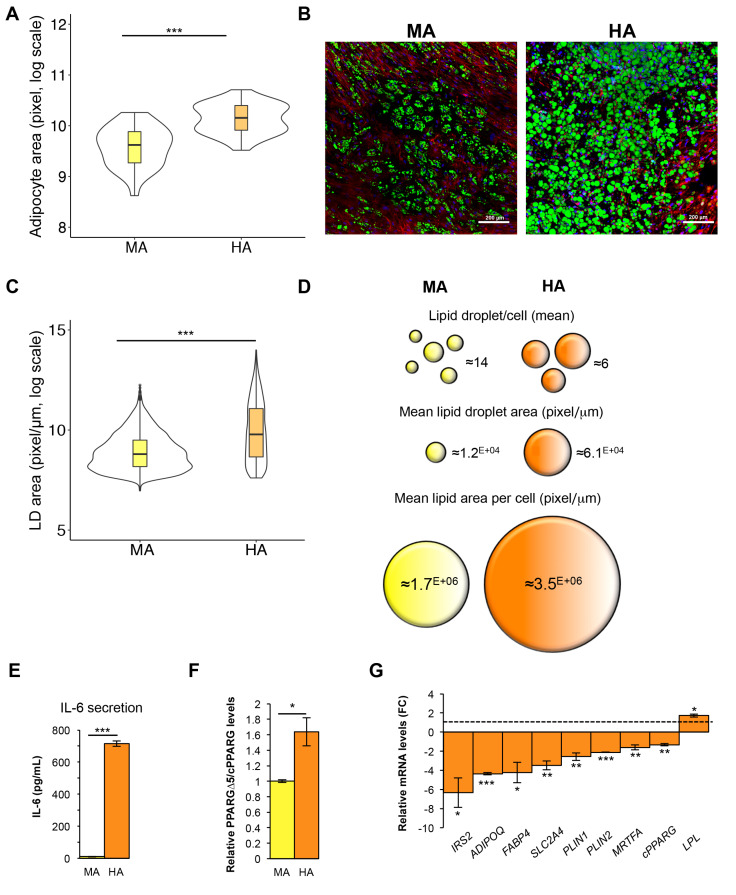
Differences between mature and hypertrophic-like adipocytes occur in cell size, lipid droplets, IL-6 secretion, and gene expression. (**A**) Violin plot showing cell area of mature (MAs) and hypertrophic-like (HAs) adipocytes (*n* = 60). *** *p* val ≤ 0.001. (**B**) Representative confocal microscopy images of hMSCs differentiated in MAs and HAs stained with DAPI (nuclei, blue), Bodipy 495/503 (lipid droplets, green) and WGA 632/647 (cell membranes, red; scale bars, 200 µm). (**C**) Violin plot showing lipid area/cell measured by 3D analysis on 2973 LDs (from 214 MAs) and on 1168 LDs (from 206 HAs). *** *p* val ≤ 0.001. (**D**) Schematic representation of 3D analysis results from LDs in MAs and HAs (i.e., mean of number of LD/cell, LD area, and total LD area/cell), as described in B. (**E**) Bar graph reporting IL-6 concentration (pg/mL) determined by ELISA on culture supernatant of MAs and HAs. Data are reported as mean ±SEM from three independent experiments. *** *p* val ≤ 0.001. (**F**) Relative PPARGΔ5/cPPARG mRNA levels (qPCR) in MAs and HAs. MAs were used as reference sample and *PPIA* as reference gene. Data are reported as mean ±SEM from three independent experiments. *** *p* val ≤ 0.001. (**G**) Relative mRNA quantifications (signed fold-changes) in HAs vs. MAs (dotted line). *PPIA* was used as reference gene. Data are reported as mean ±SEM from three independent experiments. * *p* val ≤ 0.05, ** *p* val ≤ 0.01 and *** *p* val ≤ 0.001.

Our in vitro model of hypertrophic-like adipocytes allows a direct comparison with mature cells. Hence, we used optical (Figure 5A) and electron (Figure 5B) microscopy for qualitative morphological and ultrastructural comparison between the two conditions. Furthermore, using confocal microscopy we evaluated cellular size as well as the number, size and distribution of LDs within adipose cells (Figure 6A–D, Figure S6B,C).

As evident in Figure 5A, HAs show a massive and diffuse staining by Oil Red O corresponding to a larger amount of neutral lipids stored in giant LDs compared with smaller LDs present in mature adipocytes. The marked enlargement of LDs in hypertrophic cells induces a substantial reduction of the cytoplasmic layer increasing the pressure on cell nucleus (Figure 3D, Figure S6A). Ultrastructure analysis reveals a different electron density of LDs between MAs and HAs suggesting a shift in the lipid content not measurable by Oil Red O staining (Figure 5B). In mature adipocytes, magnifications show contacts between LDs and mitochondria, well-structured ER nearby LDs and LD–LD contacts indicating fusion sites between coalescent LDs (Figure 5B, upper panels). In hypertrophic-like cells, large LDs are surrounded by several mitochondria and an extensive network of intermediate filaments (IFs; Figure 5B, lower panels). Particularly, in line with previous studies on differentiating 3T3-L1 [47] and mouse macrophage foam cells [48], IFs form cage-like structures around LDs (Figure 5B, lower panel). These structures may serve both to prevent contacts with other droplets or organelles [49] and to regulate lipids influxes and effluxes [47,50], especially in hypertrophic cells with large LDs.

Estimation of cell size by confocal microscopy revealed a marked increase of cellular area (FC~1.7) in hypertrophic-like cells compared to mature ones (Figure 6A, Figure S6B). A direct comparison of lipid accumulation in mature and hypertrophic-like cells revealed a global increase (~35%) in lipid accumulation in HAs vs. MAs (Figure 5A). Interestingly, LDs analysis by confocal microscopy revealed a marked increase in the mean area (FC~5), and a doubling of lipid content, with a reduction (~41%) of total LD number (Figure 6B–D, Figure S6C). Overall, this analysis supports the formation of giant LDs by both increased lipid accumulation and coalescence of small droplets [51,52], suggesting that these processes govern the transformation of LDs during the transition of human adipocytes to hypertrophic state. Additionally, in line with the work of Skurk and colleagues (2007) [53], in vitro-generated HAs secrete higher levels of IL-6 compared to mature adipocytes (Figure 6E). Among the well-characterized cytokines released by hypertrophic adipocytes, IL-6 is the most studied due to its role in defective adipogenesis and IR onset [54,55,56,57].

Noteworthy, mirroring what observed in the SAT of hypertrophic obese patients, HAs display reduced levels of cPPARG mRNA and increased PPARGΔ5/cPPARG ratio (Figure 6F). Despite cPPARG expression being only modestly reduced in HAs (vs. MAs), PPARγ target genes are highly reduced, compatibly with the impaired metabolic activity in hypertrophic AT (Figure 6G). Hence, in light of the dominant negative activity of PPARGΔ5 [16], we speculate that increased PPARGΔ5/cPPARG ratio in hypertrophic adipose cells may contribute—at least in part—to inhibit the transcription of direct PPARγ target genes. Differently, despite *LPL* being a PPARγ target, its expression in HAs is increased compared to mature cells. However, this is in line with high *LPL* levels in SAT of obese patients [58] and the elevated enzymatic activity in hypertrophic AT [59,60]. Furthermore, insulin and rosiglitazone (contained in the AMM) are likely to promote *LPL* increase during the in vitro transition from mature to hypertrophic-like adipose cells [61]. Genes encoding proteins involved in insulin signaling and associated with insulin sensitivity, such as *IRS2* and *SLC2A4*, were among the most down-regulated PPARγ targets in hypertrophic cells, in line with the notion that IR is a hallmark of hypertrophic obesity. Since perlipin 1 can restrain the pro-inflammatory response—reducing futile lipolysis—and promote insulin sensitivity [62], *PLIN1* gene expression is strongly down-regulated in hypertrophic-like cells compared to matureones. The pathologic transition of adipocytes toward the hypertrophic state also induces a marked decrease of *MRTFA* expression (Figure 6G, Figure S5E), whereas cPPARG—albeit reduced (Figure 6G)—is still highly expressed in these cells (Figure 4B, Figure S5E), in line with its antagonist activity toward MRTFA in adipose cells.

### 3.6. GLUT4 Negatively Correlates with PPARGΔ5/cPPARG Ratio Only in Pathologic Conditions

PPARγΔ5 has a proven dominant negative activity on canonical PPARγ and is highly expressed in human SAT [16]. In light of this evidence, it is reasonable to consider that the global PPARγ activity in this tissue is tightly dependent on the relative amount between its canonical and dominant negative isoforms. As evidenced by the comparison between mature and hypertrophic-like adipose cells, we observed the unbalance between *PPARG* isoforms (increased PPARGΔ5/cPPARG ratio) and a concomitant pronounced alteration—in terms both of mRNA and protein expression—of PPARγ target genes involved in insulin signaling, and particularly of *SLC2A4* encoding the inducible glucose transporter 4 (Figure 4B–C). Accordingly, even in vivo large adipocytes display unbalanced PPARGΔ5/cPPARG ratio (Figure 1D and Figure 6E), whose increase negatively impacts PPARγ transactivation ability in vitro [16].

**Figure 7:**
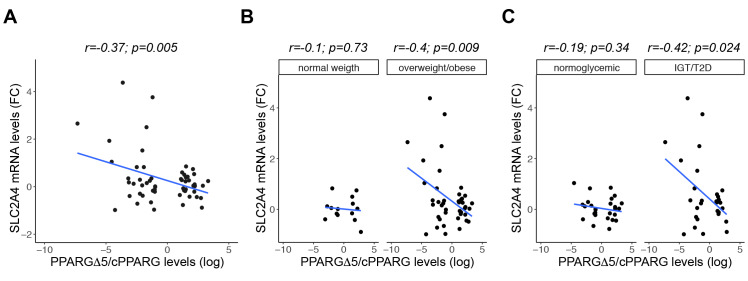
PPARGΔ5/cPPARG ratio correlates with *SLC2A4* levels. PPARGΔ5 and cPPARG expression was previously measured in Aprile et al. (2018). (**A**–**C**) Scatterplot reporting the correlations by linear regression analysis between *SLC2A4* and PPARGΔ5/cPPARG levels (qPCR) in the SAT of a subset of individuals (*n* = 56), stratified in subgroups according to BMI in normal weight (*n* = 14) and overweight/obese (*n* = 42), or to glucose-metabolizing capacity in NGT (*n* = 27), IGT and T2D (*n* = 29). *RPS23* was used as reference gene. Pearson’s correlation coefficient (r) and *p* values (*p*) are shown.

Hence, we tested the hypothesis of a correlation between PPARGΔ5/cPPARG ratio and *SLC2A4* expression in vivo, measuring mRNA levels in the SAT of a subset of individuals from our German cohort (*n* = 56). As shown in Figure 7A, *SLC2A4* negatively correlates with PPARGΔ5/cPPARG ratio in the entire cohort, whereas cPPARG expression shows an opposite trend (Figure S7A). Interestingly, we disclosed significant correlation only in obese/overweight individuals (vs. individuals with normal weight; Figure 7B and Figure S7B), as well as in patients with altered glucose metabolism, i.e., IGT and T2D (vs. individuals with normal glucose tolerance, NGT; Figure 7C; Supplementary Figure S7C). PPARγ-mediated induction of *GLUT4* is a primary mechanism to establish insulin sensitivity of adipose tissue, liver and skeletal muscle [45,46,63]. Therefore, these data suggest that the unbalance of *PPARG* isoforms in the SAT—and particularly high PPARGΔ5/cPPARG ratio—can further contribute to IR onset in the adipose tissue of patients with hypertrophic obesity.

## 4. Conclusions

Inappropriate expansion of adipocytes in the SAT is a characteristic of hypertrophic obesity, associated with a reduced adipogenesis and impaired insulin sensitivity. These primary events contribute to establish local inflammation and reduced insulin sensitivity in the AT, leading to ectopic fat deposition and systemic IR [5,6,7,8]. Hence, adipocyte size has been proposed as a predictor of IR and T2D onset. Nevertheless, technical drawbacks, the dynamical distribution of adipose cells in AT, and inter-individual variability make difficult accurately determining adipocyte size and establishing a value (or a range) that is indicative of metabolically defective cells. Then, adequate cellular models recapitulating the physiological aspects of neo-adipogenesis and the pathological features of hypertrophic metabolically unhealthy adipocytes are needed for addressing factors responsible of the AT shift toward the hypertrophic state.

Our recent work established PPARγΔ5 - a dominant negative isoform of PPARγ - as a potential contributor to the functional PPARγ impairment in the SAT of obese patients [16]. Our previous data suggest that the unbalanced ratio between dominant negative and canonical isoforms in the SAT can contribute to the transcriptional repression of metabolic genes and to the impairment of neo-adipogenesis. Both these pathologic features are hallmarks of hypertrophic obesity and are strictly related to IR and T2D onset [1,42,44]. It prompted us exploring whether *PPARG* splicing is affected in the context of hypertrophic obesity. Our in vivo finding that the ratio between PPARGΔ5 and canonical *PPARG* transcripts is significantly higher in SAT enriched of large adipocytes corroborates the finding that PPARγ activity is impaired in adipose tissue when reduced insulin sensitivity and defective neo-adipogenesis are in place. Of note, the observation that canonical and dominant negative *PPARG* transcripts have opposite correlation‚ not only with BMI and body fat [16], but also with LDL-cholesterol and leptin serum levels, further highlights the differential role of *PPARG* isoforms in the SAT.

However, our interest in studying in a more controlled and unbiased system, whether *PPARG* expression and splicing are affected in hypertrophic adipocytes guided us to set-up a new cellular model of human adipocyte hypertrophy through the generation of hypertrophic-like cells directly comparable to mature ones. Low variability in the differentiation rate, together with a weak susceptibility to dedifferentiate and detach in culture, represent only some advantages of this model. Indeed, by a detailed morphological, ultrastructural and transcriptional analysis, we provide a qualitative and quantitative estimation of the differentiation process, ranging from hMSCs to mature adipocytes as well as of the pathological shift toward the hypertrophic state. Indeed, the transition from terminally differentiated to hypertrophic-like cells mimics the dynamics of hypertrophic AT, showing the genesis of giant LDs—surrounded by an extensive network of IFs and mitochondria - which induce progressive cell engulfment, thickening of the cytoplasmic layer and increased pressure on cell nuclei. An accurate analysis of lipid droplets provided a quantitative estimation of lipid accumulation in hypertrophic-like cells, supporting the model in which giant LDs originate either by progressive lipid storage in single LDs and by coalescence of smaller droplets. Beyond the morphological changes, we also observed that hypertrophic-like adipocytes secrete high amount of IL-6, a pro-inflammatory cytokine typically observed in the microenvironment of hypertrophic AT. Notably, higher IL-6 levels are secreted by hypertrophic-like adipocytes compared to normal cells, further supporting the bona fide of this new cellular model. Along with the progressive lipid accumulation and the following morphological changes, we also systematically explored how these cells modulate gene/protein expression. The finding that throughout the differentiation of hMSCs several PPARγ target genes involved in LDs formation, insulin signaling and lipid metabolism display peculiar expression patterns compatible with PPARγ levels, further supports the use of hMSCs as model of human adipogenesis. Additionally, the progressive switch in the expression of perilipin genes—compatible with the replacement of ADRP with perilipin 1 in LDs formation—and the opposite trend of expression for *MRTFA* and *PPARG*, consistent with their supposed mutual antagonistic activity, are in line with previous studies in murine cells [38,41]. Mimicking the pathologic state of human AT, hypertrophic-like cells display a marked increase of PPARGΔ5/cPPARG ratio and a substantial reduction of PPARγ target genes involved in LDs biogenesis and insulin signaling, especially of Glut4. We found a significant negative correlation between PPARGΔ5/cPPARG ratio and *SLC2A4* expression only in patients with overweight/obesity as well as in those having altered glucose metabolism (i.e., with impaired glucose tolerance or T2D), but not in individuals with normal BMI or with normal glucose tolerance. These in vitro and in vivo data support the hypothesis that the unbalance between *PPARG* canonical and dominant negative isoforms is a characteristic of hypertrophic adipocytes, and that it associates with a marked perturbation of the PPARγ-dependent gene network, with a pronounced down-regulation of factors involved in glucose and lipid metabolism, such as Glut4. Furthermore, beyond the in vivo investigation of *PPARG* isoforms in hypertrophic AT, to the best of our knowledge, here we describe the first in vitro model of human hypertrophic-like adipocytes. This cellular model can be instrumental for dissecting—in the absence of confounding effects—the molecular mechanisms underlying the functional defects of the adipocytes in hypertrophic AT. Indeed, the use of a unique in vitro model—able to recapitulate each differentiation step from hMSCs to mature adipocyte and further toward hypertrophic state—is a powerful tool to decipher in a stepwise manner the pathological determinants of AT dysfunction in obesity and in its related comorbidities.

## Figures and Tables

**Table 1:** Characteristics of the study participants.

	Women	Men
***n***	51	35
**Age (years)**	53.4 ± 16.6	58.4 ± 16.3
**Body weight (kg)**	99.5 ± 34.4	109 ± 44.9
**BMI (kg/m²)**	36.5 ± 12	34.1 ± 12.3
**Body fat (%)**	35 ± 11.7	30.7 ± 9.5
**Visceral fat area (cm²)**	165.5 ± 119.6	172.9 ± 134
**Subcutaneous fat area (cm²)**	471 ± 492.5	418.5 ± 333.3
**Waist circumference (cm)**	114 ± 33.1	120.3 ± 24
**FPG (mmol/L)**	5.9 ± 1.2	5.7 ± 0.9
**FPI (pmol/L)**	127.5 ± 133.1	81.4 ± 89.8
**HbA1c (%)**	5.9 ± 0.8	5.9 ± 0.62
**Clamp GIR (µmol/kg/min)**	79 ± 35	75.2 ± 32
**Cholesterol (mmol/L)**	5.1 ± 0.75	4.9 ± 1.02
**HDL-Cholesterol (mmol/L)**	1.2 ± 0.3	1.1 ± 0.3
**LDL-Cholesterol (mmol/L)**	3.6 ± 1.2	3.5 ± 1.2
**Triglycerides (mmol/L)**	1.42 ± 0.36	1.9 ± 1.6
**Free fatty acids (mmol/L)**	0.44 ± 0.38	0.47 ± 0.4
**hsCRP (mg/L)**	12.3 ± 14.8	11.5 ± 14.1
**IL-6 (pg/mL)**	4.2 ± 4.1	3.3 ± 4.4
**ALAT (µkat/L)**	0.8 ± 1.1	0.67 ± 0.7
**ASAT (µkat/L)**	0.7 ± 0.85	0.63 ± 0.5
**GGT (µkat/L)**	1.9 ± 3.5	1.5 ± 2.5
**Adiponectin (µg/mL)**	9.1 ± 6.6	5 ± 3**
**Leptin (pg/mL)**	40 ± 20	18.5 ± 11.5**
**Mean subcutaneous adipocyte diameter (µm)**	110.6 ± 11.6	107.6 ± 9.3
**Mean visceral adipocyte diameter (µm)**	100 ± 7.4	97.6 ± 5.7

Data are means ± SD. ** *p* < 0.01 for gender differences. Abbreviations: ALAT—Alanine-Aminotransferase; ASAT—Aspartate-Aminotransferase; BMI—body mass index; FPG—fasting plasma glucose; FPI—fasting plasma insulin; GGT—gamma- glutamyl transpeptidase; HbA1c—glycated haemoglobin; HDL—high density lipoproteins; hsCRP—high sensitivity C-reactive protein; IL-6—Interleukin 6; LDL—low density lipoproteins.

## Supplementary Materials

The following are available online at https://www.mdpi.com/2073-4409/9/5/1284/s1, Figure S1. Correlation of PPARGΔ5 and cPPARG levels with clinical and biochemical parameters; Figure S2. Adipocyte differentiation of hMSCs is affected by densities of cell plating; Figure S3. Stemness markers expression in terminally differentiated hMSCs and in hypertrophic-like cells; Figure S4. Hypertrophic-like adipocytes are less prone to dedifferentiation in vitro; Figure S5. Expression trends of PPARγ and its target genes; Figure S6. Morphometric characteristics of hypertrophic-like adipocytes; Figure S7. The expression of canonical *PPARG* transcripts correlates with *SLC2A4* levels; Table S1. Sequences of oligonucleotides used in RT-PCR and qPCR assays; Table S2. Reagents and resources information.
