# Supplementary material for: In Vitro-Generated Hypertrophic-Like Adipocytes Displaying PPARG Isoforms Unbalance Recapitulate Adipocyte Dysfunctions In Vivo
Source: Cells. 2020 May 21;9(5):1284. doi: 10.3390/cells9051284 (PMC7290899; doi:10.3390/cells9051284)
Supplement: Supplementary file 1 [file cells-09-01284-s001.pdf]

Figure S1

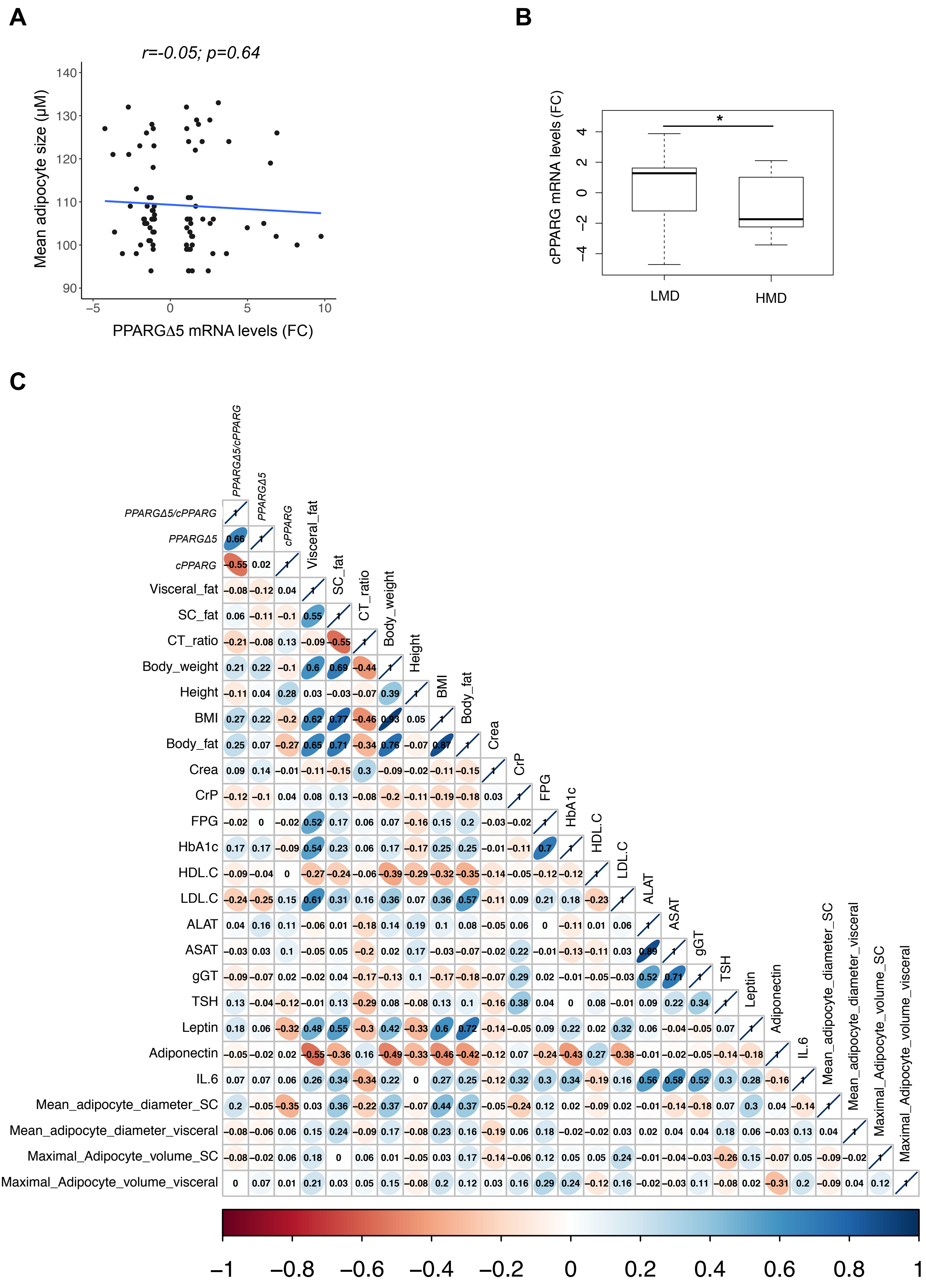

A
r=-0.05; p=0.64
Mean adipocyte size (µM)
140
130
120
110
100
90
−5
0
5
10
PPARGΔ5 mRNA levels (FC)
B
cPPARG mRNA levels (FC)
4
2
0
−2
−4
*
LMD
HMD
C
PPARGΔ5/cPPARG 1
PPARGΔ5 0.66 1
cPPARG −0.55 0.02 1
Visceral_fat −0.08 −0.12 0.04 1
SC_fat 0.06 −0.11 −0.1 0.55 1
CT_ratio −0.21 −0.08 0.13 −0.09 −0.55 1
Body_weight 0.21 0.22 −0.1 0.6 0.69 −0.44 1
Height −0.11 0.04 0.28 0.03 −0.03 −0.07 0.39 1
BMI 0.27 0.22 −0.2 0.62 0.77 −0.46 0.93 0.05 1
Body_fat 0.25 0.07 −0.27 0.65 0.71 −0.34 0.76 −0.07 0.87 1
Crea 0.09 0.14 −0.01 −0.11 −0.15 0.3 −0.09 −0.02 −0.11 −0.15 1
CrP −0.12 −0.1 0.04 0.08 0.13 −0.08 −0.2 −0.11 −0.19 −0.18 0.03 1
FPG −0.02 0 −0.02 0.52 0.17 0.06 0.07 −0.16 0.15 0.2 −0.03 −0.02 1
HbA1c 0.17 0.17 −0.09 0.54 0.23 0.06 0.17 −0.17 0.25 0.25 −0.01 −0.11 0.7 1
HDL.C −0.09 −0.04 0 −0.27 −0.24 −0.06 −0.39 −0.29 −0.32 −0.35 −0.14 −0.05 −0.12 −0.12 1
LDL.C −0.24 −0.25 0.15 0.61 0.31 0.16 0.36 0.07 0.36 0.57 −0.11 0.09 0.21 0.18 −0.23 1
ALAT 0.04 0.16 0.11 −0.06 0.01 −0.18 0.14 0.19 0.1 0.08 −0.05 0.06 0 −0.11 0.01 0.06 1
ASAT −0.03 0.03 0.1 −0.05 0.05 −0.2 0.02 0.17 −0.03 −0.07 −0.02 0.22 −0.01 −0.13 −0.11 0.03 0.89 1
gGT −0.09 −0.07 0.02 −0.02 0.04 −0.17 −0.13 0.1 −0.17 −0.18 −0.07 0.29 0.02 −0.01 −0.05 −0.03 0.52 0.71 1
TSH 0.13 −0.04 −0.12 −0.01 0.13 −0.29 0.08 −0.08 0.13 0.1 −0.16 0.38 0.04 0 0.08 −0.01 0.09 0.22 0.34 1
Leptin 0.18 0.06 −0.32 0.48 0.55 −0.3 0.42 −0.33 0.6 0.72 −0.14 −0.05 0.09 0.22 0.02 0.32 0.06 −0.04 −0.05 0.07 1
Adiponectin −0.05 −0.02 0.02 −0.55 −0.36 0.16 −0.49 −0.33 −0.46 −0.42 −0.12 0.07 −0.24 −0.43 0.27 −0.38 −0.01 −0.02 −0.05 −0.14 −0.18 1
IL.6 0.07 0.07 0.06 0.26 0.34 −0.34 0.22 0 0.27 0.25 −0.12 0.32 0.3 0.34 −0.19 0.16 0.56 0.58 0.52 0.3 0.28 −0.16 1
Mean_adipocyte_diameter_SC 0.2 −0.05 −0.35 0.03 0.36 −0.22 0.37 −0.07 0.44 0.37 −0.05 −0.24 0.12 0.02 −0.09 0.02 −0.01 −0.14 −0.18 0.07 0.3 0.04 −0.14 1
Mean_adipocyte_diameter_visceral −0.08 −0.06 0.06 0.15 0.24 −0.09 0.17 −0.08 0.23 0.16 −0.19 0.06 0.18 −0.02 −0.02 0.03 0.02 0.04 0.04 0.18 0.06 −0.03 0.13 0.04 1
Maximal_Adipocyte_volume_SC −0.08 −0.02 0.06 0.18 0 0.06 0.01 −0.05 0.03 0.17 −0.14 −0.06 0.12 0.05 0.05 0.24 −0.01 −0.04 −0.03 −0.26 0.15 −0.07 0.05 −0.09 −0.02 1
Maximal_Adipocyte_volume_visceral 0 0.07 0.01 0.21 0.03 0.05 0.15 −0.08 0.2 0.12 0.03 0.16 0.29 0.24 −0.12 0.16 −0.02 −0.03 0.11 −0.08 0.02 −0.31 0.2 −0.09 0.04 0.12 1
−1 −0.8 −0.6 −0.4 −0.2 0 0.2 0.4 0.6 0.8 1

Figure S1. Correlation of PPARGΔ5 and cPPARG levels with clinical and biochemical parameters

(A) Scatterplot resulting from regression analysis and showing correlation between PPARGΔ5 levels and mean diameter of subcutaneous adipocyte size (N=86). Pearson correlation coefficient (r) and p value (p) are shown.

(B) Boxplot showing cPPARG levels in two subgroups, defined according to the mean diameter of subcutaneous adipocytes as “Low Mean Diameter” (LMD; mean diameter <115μm, N=63) and “High Mean Diameter” (HMD; mean diameter >115μm, N=23) group. *$p<0.05$.

(C) Corrplot reporting the Pearson’s correlation coefficients for cPPARG, PPARGΔ5, PPARGΔ5/cPPARG and several clinical and biochemical parameters. CT_ratio=VAT/SAT ratio by computing tomography, CREA=Creatinine, CRP=C-reactive protein, FPG=fasting plasma glucose, HbA1c=Hemoglobin A1c, HDL.C=High-Density Lipoprotein-Cholesterol, LDL.C=Low-Density Lipoprotein-Cholesterol, ALAT=Alanine aminotransferase, ASAT=Aspartate transaminase, gGT=gamma-glutamyl transpeptidase, SC=subcutaneous.

Figure S2

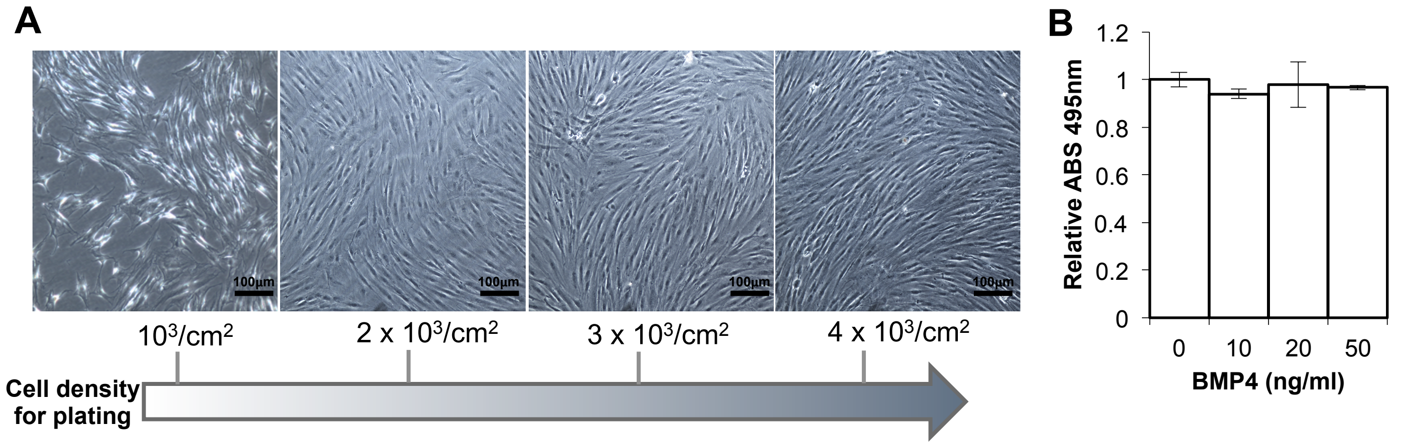

Figure S2. Adipocyte differentiation of hMSCs is affected by densities of cell plating

(A) Representative phase-contrast images of hMSCs plated at different densities (10^3, $2x10^3$, $3x10^3$ and $4x10^3/cm^2$; scale bars, 100 μm).

(B) Lipid accumulation by oil Red O staining (optical density) of mature adipocytes differentiated from hMSCs with adipogenic mixes supplemented with increasing doses (i.e. 10, 20 and 50 ng/mL) of Bone Morphogenic Protein 4 (BMP4). Data are reported as mean ±SEM *vs* cells treated with vehicle (0 ng/mL).

Figure S3

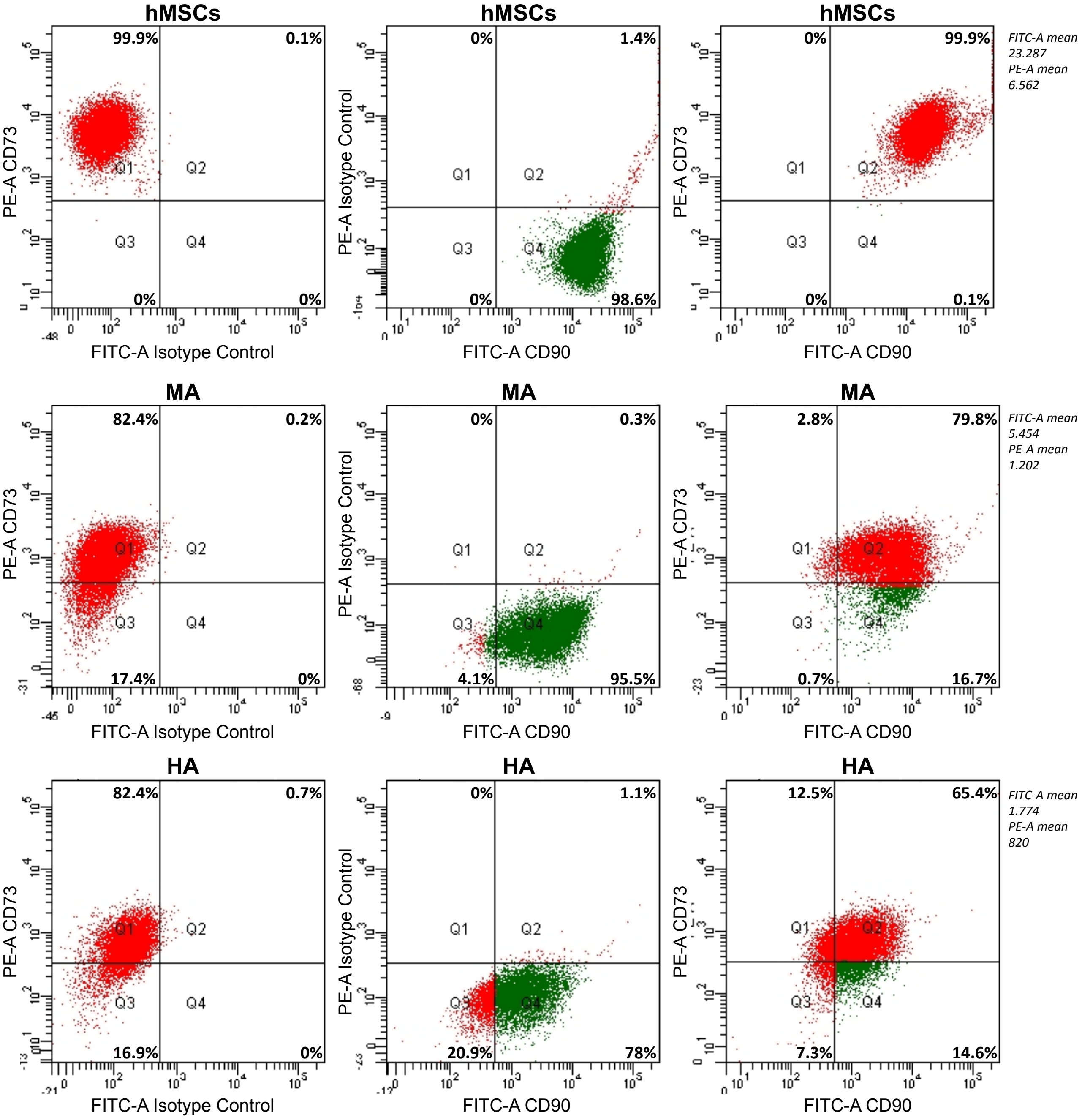

Figure S3. Stemness markers expression in terminally differentiated hMSCs and in hypertrophic-like cells

Flow cytometry analysis of CD73 and CD90 markers in hMSCs at starting point (hMSCs), in mature (MA) and hypertrophic-like adipocytes (HA). PE and FITC fluorophores were conjugated to anti-CD73 and anti-CD90 antibodies, respectively. Left boxes show expression values of FITC- and PE-isotype controls.

Figure S4

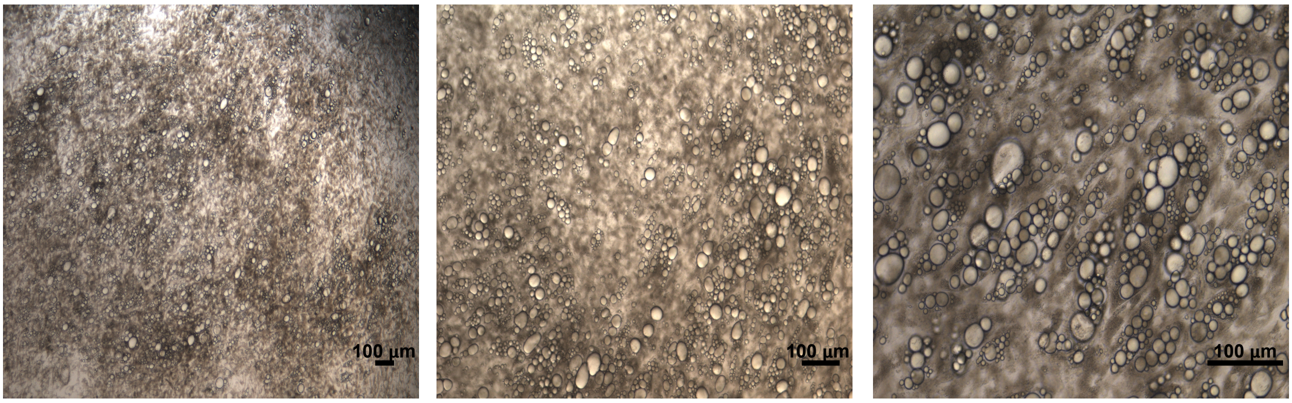

5x magnification   10x magnification   20x magnification

Figure S4. Hypertrophic-like adipocytes are less prone to dedifferentiation *in vitro*
Representative phase-contrast images at different magnifications cultured of hypertrophic-like adipocytes in standard conditions for additional 30 days (scale bars, 100 µm).

Figure S5

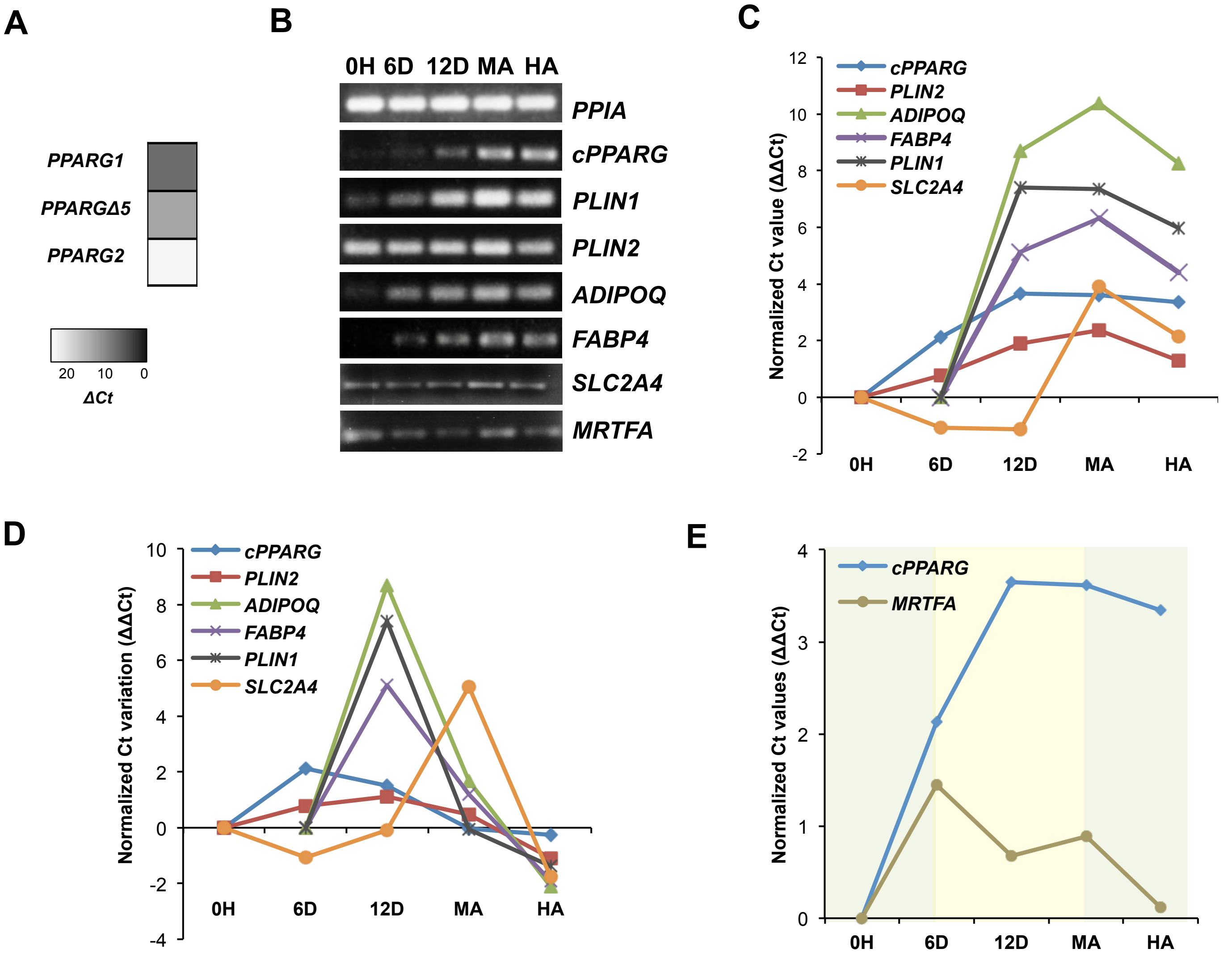

Figure S5. Expression trends of PPARγ and its target genes

(A) Gray-scale heatmap of normalized mRNA expression values (i.e. ΔCt= Ct gene - Ct *PPIA*) of canonical (PPARG1 and PPARG2) and dominant negative (PPARGΔ5) *PPARG* transcripts determined by qPCR in hMSCs (T=0 hours). *PPIA* was used as reference gene.

(B) Representative gel images of RT-PCR assays in hMSCs at different time points upon adipogenic induction (T=0 hours, T=6 and T=12 days) and in mature and hypertrophic-like adipocytes (MAs and HAs, respectively).

(C) Line charts displaying gene expression trends (normalized mRNA expression values; ΔΔCt= ΔCt sample-ΔCt reference sample) measured by qPCR. *PPIA* was used as reference gene for all analyzed genes. For each gene, the first time point showing detectable mRNA levels was used as reference (ΔΔCt=0). Data are reported as mean from three independent experiments.

(D) Line charts showing gene expression variations between two subsequent time points (ΔΔCt time point 2 - ΔΔCt time point 1) along hMSCs differentiation determined by qPCR. *PPIA* was used as reference gene and data are reported as mean from three independent experiments.

(E) Line charts comparing gene expression trends - analyzed as in panel C - of cPPARG and *MRTFA* genes along hMSCs differentiation. Similar expression trends at specific time points are indicated by light green boxes (from T=0 hours to T=6 days, from MA to HA) and opposite trends in stages indicated by yellow box (from T=6 days to MA).

Figure S6

A

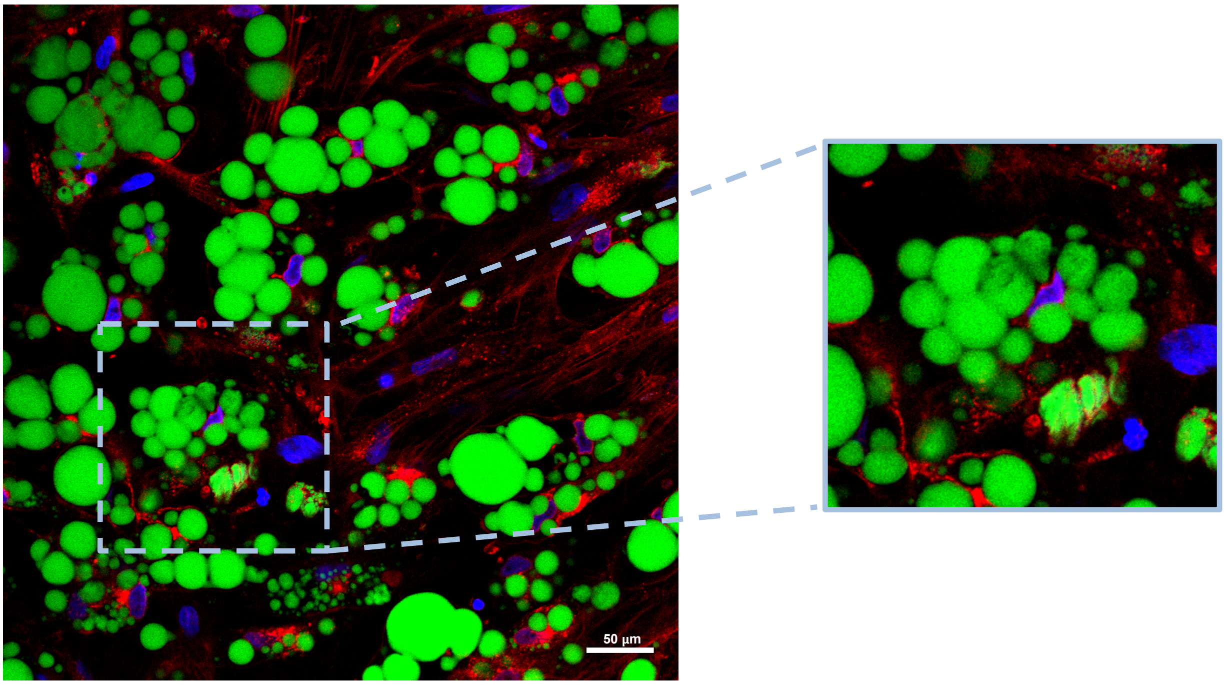

B

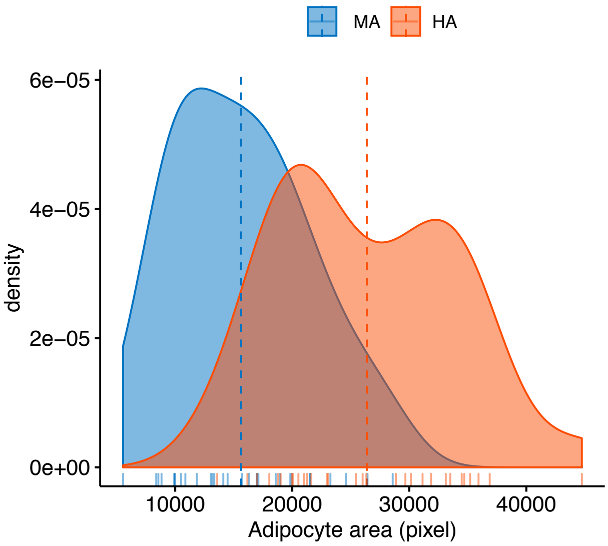

C

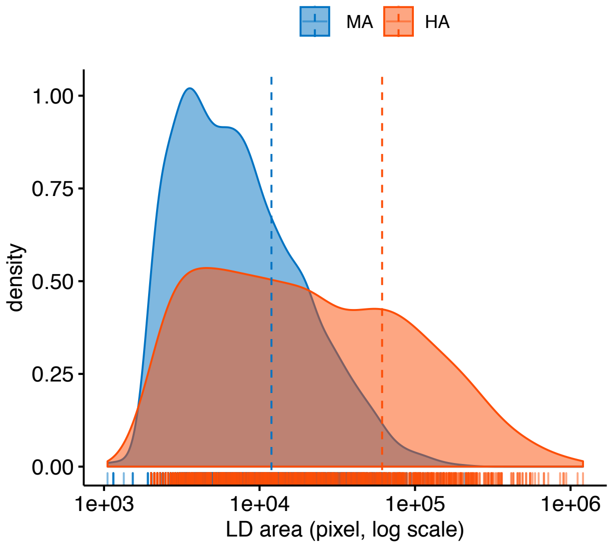

Figure S6. Morphometric characteristics of hypertrophic-like adipocytes
(A) Representative confocal microscopy images displaying a marked *nuclei* pressure in hypertrophic-like adipocytes stained with DAPI (*nuclei*, blue), Bodipy 495/503 (lipid droplets, green) and WGA 632/647(cell membranes, red; scale bar, 50 μm).
(B-C) Density plots showing value distribution of (B) adipocyte area - analyzed on MAs (n=30) and HAs (n=30) - and (C) lipid droplets area measured on 2973 LDs (from 214 MAs) and on 1168 LDs (from 206 HAs).

Figure S7

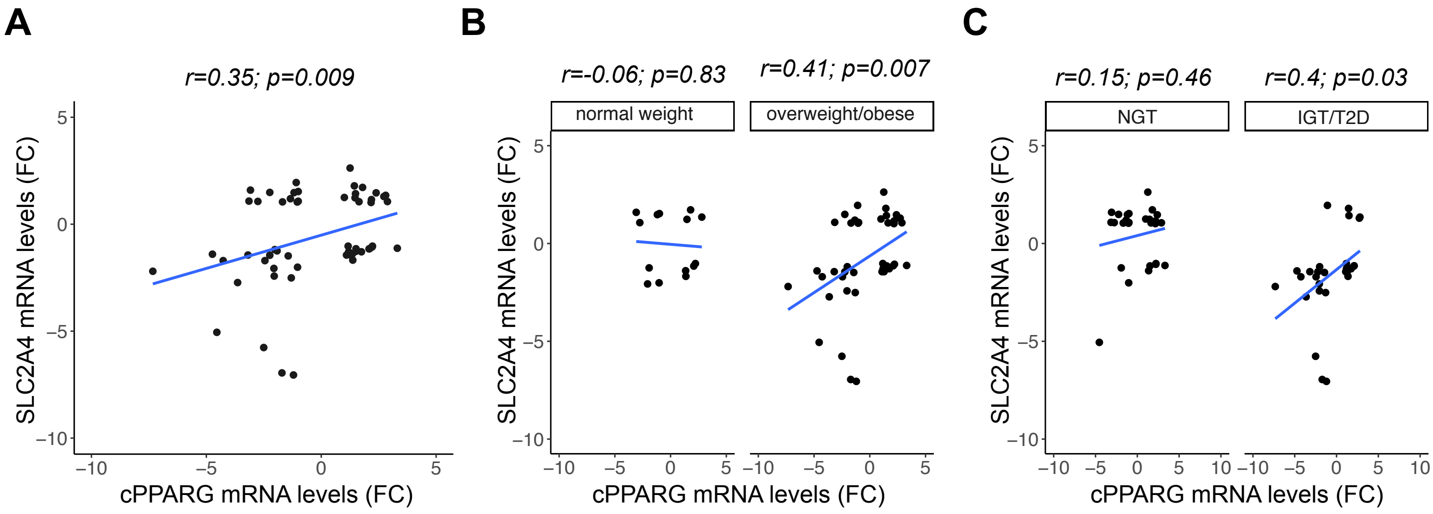

Figure S7. The expression of canonical *PPARG* transcripts correlates with *SLC2A4* levels

cPPARG expression was previously measured in Aprile et al., 2018.

(A-C) Scatterplot reporting the correlation by linear regression analysis between *SLC2A4* and cPPARG expression levels (qPCR) in the SAT of a subset of individuals (N=56); stratified in subgroups according to BMI in normal weight (N=14) and overweight/obese (N=42), or to glucose-metabolizing capacity in NGT (N=27), IGT and T2D (N=29). *RPS23* was used as reference gene. Pearson's correlation coefficient (r) and p values (p) are shown.

Table S1. Sequences of oligonucleotides used in RT-PCR and qPCR assays

Gene	Sense Primer (5′-3′)	Antisense Primer (5′-3′)	Melting Temperature (°C)	PCR product (bp)	Reference figure
cPPARG	GAGAAGGAGAAGCTGTTGGC	ATGGCCACCTCTTTGCTCT	60	272	1B-C-D; 3C; 4A-B; 6F-G; 7A-B-C; S5B-C-D-E; S7A-B-C
ADIPOQ	CTGGTGAGAAGGGTGAGAAA	GTTCTCCTTTCCTGCCTTGG	60	126	3C; 4A-B; 6G; S5B-C-D
FABP4	TGTGTGATGCTTTTGTAGGTAC	CTTCGTCAAATTCCTGGCCC	60	215	3C; 4A-B; 6G; S5B-C-D
LPL	CGCCGCCGACCAAAGAAGA	AGGTAGCCACGGACTCTGC	60	122	4A-B; 6G
PPARGΔ5	CTTGCAGTGGGGATGTCTCA	CAGCAAACCTGGGCGGTTGA	60	242	1A-C-D; 6F-G; S5A
PPIA	TACGGGTCCTGGCATCTTGT	GGTGATCTTCTTGCTGGTCT	60	196	3C; 4A-B; 6F-G; S5A-B-C-D-E
MRTFA	TTGAAACTCCAGCAGCGCC	GCTCCTTCTCTGCTCATGA	60	99	4A-B; 6G; S5B-E
SLC2A4	CGTCGGGCTTCCAACAGAT	GAGCCAAGCACCGCAGAGA	60	100	3C; 4A-B; 6G; 7A-B-C; S5B-C-D; S7A-B-C
PLIN1	GCCTCACCTTGCTGGATGG	GTGGGCTTCCTTAGTGCTG	60	128	3C; 4A-B; 6G; S5B-C-D
PLIN2	GGGCTAGACAGGATTGAGGA	TCACTGCCCCTTTGGTCTTG	60	181	3C; 4A-B; 6G; S5B-C-D
IRS2	TACATCGCCATCGACGTGAG	TCAATGCTGGCGTAGGTGTT	60	215	3C; 4A-B; 6G
RPS23	TCGTGGACTTCGTACTGCT	GCTGTGATTTTCTTGCCATTC	60	237	1A-B-C-D; 7A-B-C; S7A-B-C
PPARG1	CGAGGACACCGGAGAGGG	TGTGTCAACCATGGTCATTTC	60	135	S5A
PPARG2	TCCATGCTGTTATGGGTGAA	TGTGTCAACCATGGTCATTTC	60	113	S5A

Table S2. Reagents and resources information

REAGENT	SOURCE	IDENTIFIER
**Antibodies**		
**PPARγ (C26H12) Rabbit mAb (N terminus)**	Cell Signaling Technology	Cat# 2435
**GLUT-4 Polyclonal Antibody**	Elabscience	Cat#E-AB-30268
**ADIPOQ Polyclonal Antibody**	Elabscience	Cat#E-AB-40301
**FABP4 Polyclonal Antibody**	Elabscience	Cat#E-AB-60028
**IRS2 Polyclonal Antibody**	Elabscience	Cat#E-AB-62281
**HSP90AA1 Mouse Monoclonal Antibody**	Origene	Cat# TA500883
**Goat Anti-Mouse IgG (H L)-HRP Conjugate**	Bio-Rad	Cat# 170-6516
**Goat Anti-Rabbit IgG (H L)-HRP Conjugate**	Bio-Rad	Cat# 170-6515
**Biological Samples**		
**Subcutaneous adipose tissue cDNA samples of a Geman cohort of patients**	Prof. Matthias Blüher. (Keller et al., 2016; Guiu-Jurado et al., 2016)	N/A
**Chemicals, Peptides, and Recombinant Proteins**		
**DMEM/F-12, no glutamine**	Thermo Fisher Scientific	Cat# 21331020
**Fetal Bovine Serum Origin: EU Approved (South American)**	Thermo Fisher Scientific	Cat# 10270106
**Fetal Bovine Serum, qualified, Australia**	Thermo Fisher Scientific	Cat# 10099141
**Penicillin-Streptomycin (5,000 U/mL)**	Thermo Fisher Scientific	Cat# 15070063
**L-Glutamine (200 mM)**	Thermo Fisher Scientific	Cat# 25030081
**BMP4 Recombinant Human Protein**	Thermo Fisher Scientific	Cat# PHC9534
**Troglitazone**	Sigma Aldrich	Cat# T2573
**Rosiglitazone**	Sigma Aldrich	Cat# R2408
**Cycloheximide**	Sigma Aldrich	Cat# 01810
**GW9662**	Sigma Aldrich	Cat# M6191
**Insulin (Humulin R)**	Lilly	Cat# AIC025707011/M
**Dexamethasone**	Sigma Aldrich	Cat# D4902
**Isobutyl-1-methylXanthine**	Sigma Aldrich	Cat# I7018
**Biotin**	Sigma Aldrich	Cat# B4639
**D- Pantothenic acid hemicalcium**	Sigma Aldrich	Cat# P5155
**Pierce IP Lysis buffer**	Thermo Fisher Scientific	Cat# 87787
**Halt Protease**	Thermo Fisher Scientific	Cat# 78429
**Phosphatase Inhibitor Cocktail**	Thermo Fisher Scientific	Cat# 78420
**Oil red O staining**	Sigma Aldrich	Cat# O1391
**Bodipy 493/503**	Thermo Fisher Scientific	Cat# D3922
**DAPI (4',6-Diamidino-2-Phenylindole, Dihydrochloride)**	Thermo Fisher Scientific	Cat# D1306
**Wheat Germ Agglutinin, Alexa Fluor™ 633 Conjugate**	Thermo Fisher Scientific	Cat# W21404
**Quick StartTM Bradford 1x Dye Reagent**	Bio- Rad	Cat# 5000205
**Pierce™ ECL Western Blotting Substrate**	Thermo Fisher Scientific	Cat# 32209
**Commercial Assays**		
**High Capacitiy cDNA Reverse Transcription kit**	Thermo Fisher Scientific	Cat# 4368814
**Experimental Models: Cell Lines**		
**hTERT immortalized Adipose Derived Mesenchymal Stem Cells**	ATCC	Cat# SCRC4000
**Software and Algorithms**		
**R Bioconductor package**	Tarazona et al., 2011	https://www.bioconductor.org/
**FACSDiva software**		https://www.bdbiosciences.com
**GelQuant.NET software**		https://www.biochemlabsolutions.com
**ImageJ**	Abramoff et al., 2004	https://imagej.net/Citing
